# Disturbed Processing of Contextual Information in HCN3 Channel Deficient Mice

**DOI:** 10.3389/fnmol.2017.00436

**Published:** 2018-01-09

**Authors:** Marc S. Stieglitz, Stefanie Fenske, Verena Hammelmann, Elvir Becirovic, Verena Schöttle, James E. Delorme, Martha Schöll-Weidinger, Robert Mader, Jan Deussing, David P. Wolfer, Mathias W. Seeliger, Urs Albrecht, Carsten T. Wotjak, Martin Biel, Stylianos Michalakis, Christian Wahl-Schott

**Affiliations:** ^1^Center for Integrated Protein Science and Center for Drug Research, Department of Pharmacy, Ludwig-Maximilians University, Munich, Germany; ^2^Neurobiochemistry of Circadian Rhythms, Department of Biology, University of Fribourg, Fribourg, Switzerland; ^3^Department of Stress Neurobiology and Neurogenetics, Max Planck Institute of Psychiatry, Munich, Germany; ^4^Institute of Anatomy, University of Zurich, Zurich, Switzerland; ^5^Department of Health Sciences and Technology, Institute of Human Movement Sciences and Sport, ETH Zurich, Zurich, Switzerland; ^6^Neuroscience Center Zurich, University of Zurich and ETH Zurich, Zurich, Switzerland; ^7^Ocular Neurodegeneration Research Group, Centre for Ophthalmology, Institute for Ophthalmic Research, Eberhard Karls University Tuebingen, Tuebingen, Germany

**Keywords:** HCN3, HCN channel, circadian rhythm, fear memory, contextual information, intergeniculate leaflet, behavior mouse, knockout mouse

## Abstract

Hyperpolarization-activated cyclic nucleotide-gated channels (HCNs) in the nervous system are implicated in a variety of neuronal functions including learning and memory, regulation of vigilance states and pain. Dysfunctions or genetic loss of these channels have been shown to cause human diseases such as epilepsy, depression, schizophrenia, and Parkinson's disease. The physiological functions of HCN1 and HCN2 channels in the nervous system have been analyzed using genetic knockout mouse models. By contrast, there are no such genetic studies for HCN3 channels so far. Here, we use a HCN3-deficient (HCN3^−/−^) mouse line, which has been previously generated in our group to examine the expression and function of this channel in the CNS. Specifically, we investigate the role of HCN3 channels for the regulation of circadian rhythm and for the determination of behavior. Contrary to previous suggestions we find that HCN3^−/−^ mice show normal visual, photic, and non-photic circadian function. In addition, HCN3^−/−^ mice are impaired in processing contextual information, which is characterized by attenuated long-term extinction of contextual fear and increased fear to a neutral context upon repeated exposure.

## Introduction

**Figure 1:**
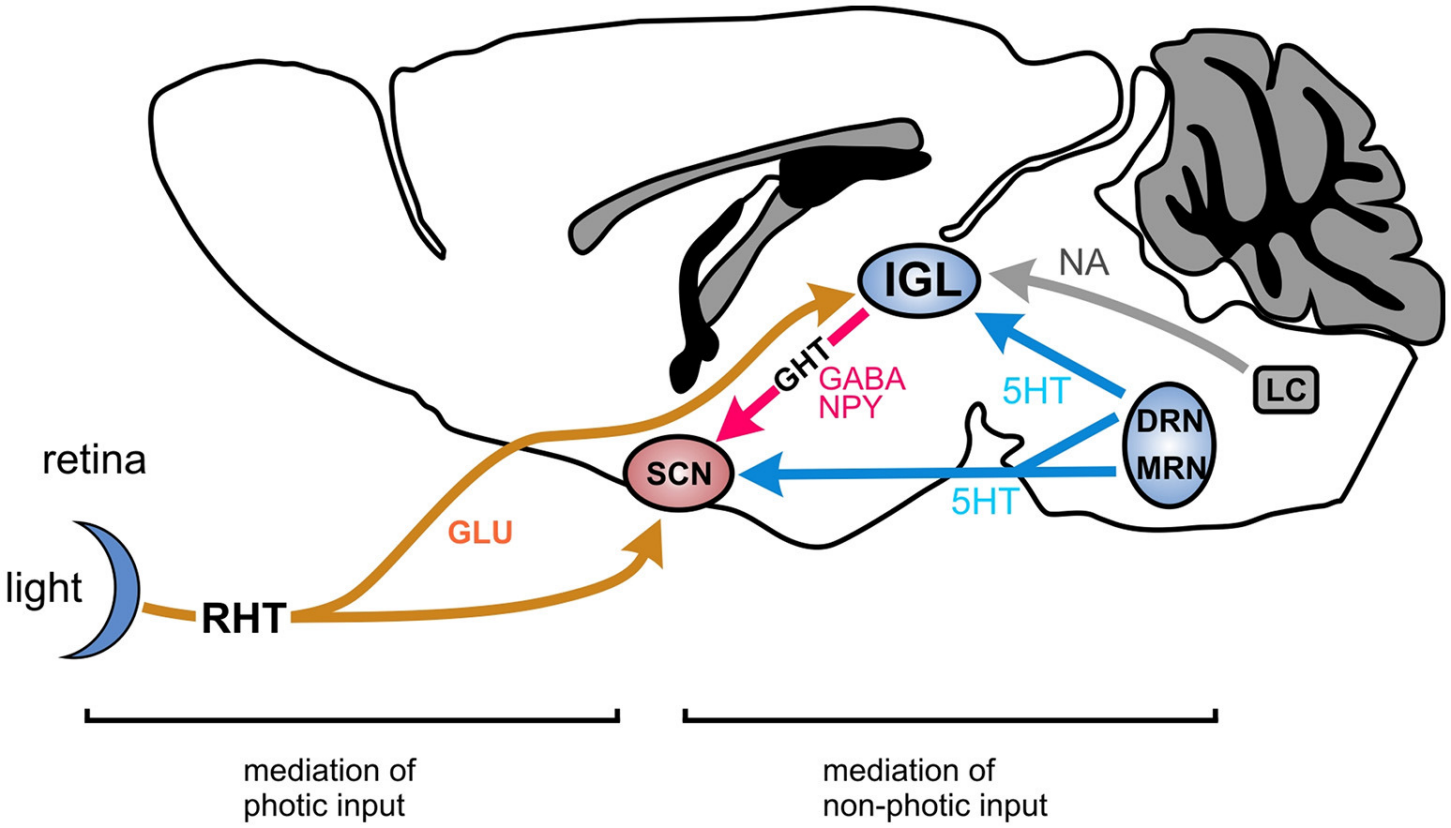
Main pathway of the circadian system in mammals involving the SCN and the IGL. The principal pathway for photic entrainment is the retinohypothalamic tract (RHT), a glutamatergic projection from the retina to the suprachiasmatic nucleus (SCN), and the intergeniculate leaflet (IGL). Non-photic entrainment cues converge on the IGL from the dorsal raphe nuclei (DRN) and the locus coeruleus (LC) and other nuclei involved in the regulation of sleep, arousal and activity. The IGL integrates these photic and non-photic inputs and conveys this information to the SCN via the geniculohypothalamic tract (GHT) which is a GABA-NPY projection. GLU, glutamate; GABA, gamma-aminobutyric acid; NPY, neuropeptide Y; 5HT, serotonin; MRN, mediane raphe nucleus.

Hyperpolarization-activated cyclic nucleotide-gated channels are widely expressed in the brain and other parts of the central and peripheral nervous systems (Pape, 1996; Robinson and Siegelbaum, 2003; Biel et al., 2009). They are particularly involved in the spatial (Nolan et al., 2004; Giocomo et al., 2011; Hussaini et al., 2011) and working memory (Wang et al., 2007), cerebellum dependent motor learning (Nolan et al., 2003), thalamic control of vigilance states (McCormick and Bal, 1997) and neuropathic pain (Emery et al., 2011). Owing to the distinct properties of these channels, different degrees of dysfunction up to a complete loss of function lead to neurological and psychiatric disorders such as epilepsy (Shah et al., 2004, 2013; Baruscotti et al., 2010; Reid et al., 2012), depression (Kim et al., 2012), and schizophrenia (Yi et al., 2016). HCN channels belong to the superfamily of voltage-gated pore loop cation channels. While they are principally operated by hyperpolarization, they are also tightly regulated by hormones and neurotransmitters that act via the second messengers cyclic AMP and cyclic GMP. The HCN channel family comprises four highly homologous members (HCN1 through HCN4). All four isoforms have been identified in the central nervous system (CNS). The current produced by these channels, termed I_h_, plays a fundamental role in controlling excitability and other electric properties of cells. In brain, I_h_ has been shown to play a key role in the control of basic functions of neurons, including determination of resting membrane potential, dendritic integration, synaptic transmission, and action potential (AP) firing (Biel et al., 2009). These functions have been attributed almost exclusively to HCN1, HCN2, and HCN4 channels. Interestingly, much less is known about the function of HCN3 channels in the CNS. The distribution of HCN3 protein in the CNS has been systematically analyzed using immunohistochemical studies in the rat where HCN3 expression was found in several brain regions including nuclei of the amygdala, paraventriular nucleus, olfactory bulb, lateral hypothalamic area, and others (Notomi and Shigemoto, 2004). So far, a few studies have attempted to elucidate functional aspects of these channels (Arroyo et al., 2006; Kanyshkova et al., 2009, 2012; Battefeld et al., 2012; Kretschmannova et al., 2012; Leist et al., 2016). Notably, in the mouse, a study postulated that HCN3 channels might be involved in the regulation of the circadian system (Ying et al., 2011). Specifically, it has been reported that HCN3 channels are present in the intergeniculate leaflet (IGL) of the hypothalamus (Notomi and Shigemoto, 2004; Ying et al., 2011). This nucleus is considered to be critical for the integration and transmission of entrainment cues to the suprachiasmatic nucleus (SCN) (Figure 1). However, no functional behavioral data have been published which investigated the role of HCN3 in the circadian system. Furthermore, expression of HCN3 has been reported in the retina (Muller et al., 2003). However, functional evidence in favor of a physiological role of HCN3 in vision is also lacking so far.

In the present study, we made use of a HCN3-deficient mouse line to experimentally address the question to which extent HCN3 channels are involved in the regulation of circadian rhythm. In addition, we explored whether HCN3 channels in the brain are determining factors for behavior.

## Materials and methods

### Ethical statement

The studies were carried out in accordance with the approved guidelines of the local committee of laboratory animal care (District Government of Upper Bavaria; Experiment allowance number 55.2-1-54-2531-88-05) and German Laws on animal welfare (Tierschutzgesetz). All procedures concerning animals adhered to the ARVO statement for the Use of Animals in Ophthalmic and Vision Research and the procedures were performed with permission of the competent government authority (Regierungspraesidium Tuebingen). Circadian experiments were done according to the law for animal protection authorized by the veterinary office of the Canton of Fribourg, Switzerland.

### Animals and housing

For the present study we used the previously published knockout mouse line, HCN3^−/−^, in which HCN3 is globally deleted (Fenske et al., 2011). This mouse line was generated using Cre/loxP-based deletion of exon 2, which encodes the first four transmembrane helices of the channel (for details see Fenske et al., 2011). After establishing the new knockout mouse line, the animals were backcrossed from a mixed C57BL6/129SV background to C57BL6/N animals for five generations before the progeny was used for testing. For this study, all experiments were performed on global HCN3^−/−^ mice and their wild type littermates whose genotypes were confirmed by PCR experiments. Unless indicated, animals were housed under an inverse 12 h light/dark cycle where lights were switched off at 7 a.m. and animals hat *ad-libitum* access to food and water.

### Immunohistochemistry

Twelve micrometer thick coronal cryosections of adult mouse brain were fixed with 4% paraformaldehyde in PBS and blocked with PBS containing 10% normal chicken serum (Vector laboratories) and 0.3% Triton X-100. Primary and secondary antibodies and dilutions used are listed below. Incubation with primary antibodies was done overnight at 4°C, and with secondary antibodies 2 h at room temperature. In experiments using horseradish peroxidase-conjugated (HRP) secondary antibodies (HCN channels), endogenous peroxidase activity was quenched (3% H_2_O_2_ in methanol) before sections were incubated with HRP antibodies. In these experiments, tyramide signal amplification was performed according to manufacturer's instruction using Cy3-conjugated tyramide (TSA-Plus Cyanine 3 System, Perkin Elmer). To visualize cell nuclei, slices were counter stained with 5 μg/ml Hoechst 33342. The brain slices were examined on an epifluorescence microscope (Axioplan 2, Zeiss) and a confocal laser scanning microscope (LSM 510, Zeiss).

### Western blot

Electrophoretic separation of proteins was done by a SDS-PAGE. Electrophoresis was performed in a mini gel apparatus (Protean 3, Biorad) at 100 V, before the gel was transferred to a tank-blot-system (Mini Trans blot, Biorad) onto a PVDF membrane (Immobilon, Millipore). After blotting (1 h, 100 V) the membrane was dried and then blocked with 5% milk powder/TBS for 1.5 h. Primary and secondary antibodies and dilutions used are listed below. Incubation with primary antibody was done overnight at 4°C. For detection, a HRP based ECL Kit (ECL system, Amersham) was used. Therefore, the membrane was incubated with HRP conjugated secondary antibody (HRP, Jackson) at RT for 1 h. The exposed films (Hyperfilm ECL, Amersham) were developed by a Curix 60 (Agfa).

### Antibodies used

#### Primary antibodies

##### Immunohistochemistry

**Table:** 

**Antibody**	**Dilution**	**Manufacturer**
rb anti-HCN3	1:1000	(Mistrik et al., 2005), #9495/1
rb anti-HCN1	1:1000	Alamone, APC-030
rb anti-HCN2	1:1000	Alamone, APC-056
rb anti-HCN4	1:500	Alamone, APC-052

##### Western blot

**Table:** 

rb anti-HCN3	1:2000	(Mistrik et al., 2005), #9495/1
ms anti-Tubulin	1:400	Dianova, MS-719-P1

#### Secondary antibodies

##### Immunohistochemistry

**Table:** 

**Antibody**	**Dilution**	**Manufacturer**
dk anti-rb HRP	1:1000	Jackson
dk anti-gp FITC	1:200	Jackson

##### Western blot

**Table:** 

dk anti-rb HRP	1:2000	Amersham
dk anti-ms HRP	1:2000	Amersham

### Preabsorption of HCN3 antibody

Unspecific binding of the HCN3 antibody was avoided by preabsorption of the antibody against HCN3^−/−^ tissue. An adult HCN3^−/−^ mouse was anesthetized and decapitated and its brain was removed, reduced to small pieces with a scalpel, and stored in a 1.5 mL Eppendorf tube. One milliliter of protease inhibitor cocktail was added and the tissue was homogenized on ice using the Ultra Turrax (T8.01, IKA-works). Ten microliters of #9495/1 HCN3-antibody were added and the mixture was agitated in an overhead shaker at 4°C for 2.5 h. Afterwards, the mixture was centrifuged at 13,400 rpm for 15 min at 4°C, the supernatant was collected into an ultracentrifugation tube and centrifuged at 30,000 rpm for 45 min. Then, sodium-azide was added to a final concentration of 0.05% and the preabsorbed antibody was stored on 4°C until usage.

### Determination of standard laboratory blood plasma parameters

For measurements of the blood plasma parameters 10 wild type and 11 HCN3^−/−^ animals were used. The mice were restrained and blood samples were collected from the lateral tail vein into lithium heparin-coated Microvette tubes (Sarstedt). Blood samples were centrifuged for 10 min at 3,000 rpm and 4°C, and 150 μl of plasma were stored on −20°C until investigation. Plasma parameters were analyzed with a Hitachi 717 autoanalyzer and reagents from Roche after diluting the plasma samples 1:2 with H_2_O as previously described (Rathkolb et al., 2000).

### Determination of corticosterone levels

The blood corticosterone levels of 10 WT and 10 HCN3^−/−^ animals were determined from animals housed under a standard 12/12h light-dark cycle where lights were switched on at 7 a.m. At 7 a.m., 1 p.m., and 7 p.m. [zeitgeber time (ZT) 0, ZT 6, and ZT 12, respectively]. One animal had to be excluded due to failure of the radioimmunoassay (RIA). Animals were anesthetized using Isoflurane (5 l/min in 95% O_2_ and 5% CO_2_) and retrobulbar blood samples were taken using heparin-coated test tubes (Kabe Labortechnik GmbH). By centrifugation (10 min, 8,500 rpm, RT) the serum was separated and stored at −80°C until further processing.

#### Corticosterone RIA

A commercially available RIA kit (ImmunoChem Double antibody Corticosterone^125^ J RIA Kit, 07-120103, MP Biomedicals for Corticosterone) was used according to manufacturer's protocol. The sensitivity of the assay is 7.7 ng/mL corticosterone and the variation was measured to be 4.4% between samples and 6.5% between runs.

### CRF concentration determination

For this experiment animals were housed under standard 12/12 h light-dark conditions with lights switched on at 7 a.m. CRF concentrations in the brain of 10 WT and 10 HCN3^−/−^ animals were determined in two groups at either 7 a.m. (ZT 0) or 7 p.m. (ZT 12). Therefore, mice were anesthetized and their whole brains - excluding the olfactory bulb - were removed and homogenized on ice in 4 mL lysis buffer using Ultra Turrax (type T8.01, IKA-Werke). Samples were centrifuged (Optima LE-80K ultracentrifuge, Beckmann Coulter) with 15,000 rpm for 20 min at RT. Then, the samples were flash-frozen on liquid nitrogen and stored on −80°C until further processing.

#### CRF-elisa

Commercially available ELISA kit (YII-YK130-EX, Cosmo Bio Co., Ltd.) was used according to manufacturer's instruction. Fluorescence was detected using a photometric optical reader (Opsys MR, Thermo Labsystems) at 490 nm. CRF contents were calculated using a standard curve (0–10 ng/mL). The sensitivity of the essay is 0.313 ng/mL for CRF. The intra-assay variation coefficient was 2.72–7.11% and inter-assay variation coefficient was 5.01–9.40%.

### Assessment of bodyweight

After weening, WT (*n* = 10) and HCN3^−/−^ (*n* = 10) animals received water and food (standard laboratory animal chow; ssniff R/M-H) *ad-libitum* and their bodyweight was determined at the age of 12 weeks.

### Electrophysiology

#### Brain slice preparation

Brain slice preparation was performed as previously described elsewhere, with slight modifications for this study (Ying et al., 2011). Briefly, HCN3 WT and HCN3^−/−^ mice (P15-P30) of either sex were anesthetized and decapitated. The head was submerged in ice-cold carbogenated (95% O_2_–5% CO_2_) slicing solution (containing in mM: 65 NaCl, 2.5 KCl, 1.25 NaH_2_PO_4_, 26 NaHCO_3_, 0.5 CaCl_2_, 7 MgCl_2_, 105 sucrose, 24.7 glucose, and 1.7 ascorbic acid) immediately. The brain was dissected out, the cerebellum was removed and the brain was then glued onto a platform. Coronal slices (300 μm) containing the intergeniculate leaflet (IGL) were prepared on a mictrotome (Microm HM 650 V, Thermo scientific) using ice-cold carbogenated slicing solution. The sections were cut into halves along the midline and transferred to carbogenated storage solution (containing in mM: 131 NaCl, 2.5 KCl, 1.25 NaH_2_PO_4_, 26 NaHCO_3_, 2CaCl_2_, 1.2 MgCl_2_, 18 glucose, 1.7 ascorbic acid, 2 sodium pyruvate and 3 myo-inositol) where they were incubated at 35°C for 30 min, and kept under room temperature for at least another hour before use.

#### Setup

Brain slices were visualized and neurons identified using a Zeiss Axioskop 2 (Jena, Germany) equipped with a 5x objective, a 4x water-immersion objective and an infrared camera (VX55, Photonics, Pittsfield, USA). Ionic currents were recorded using an EPC 10 amplifier and PatchMaster software. The IGL structure and recording sites were identified using standard mouse brain atlas (Franklin and Paxinos, 2007).

#### I_h_-recordings

Whole-cell voltage-clamp recordings were performed at 32°C. Slices were perfused with carbogenated artificial cerebrospinal fluid (aCSF; containing in mM: 131 NaCl, 2.5 KCl, 1.25 NaH_2_PO_4_, 26 NaHCO_3_, 2CaCl_2_, 1.2 MgCl_2_, 18 glucose, and 1.7 ascorbic acid). Ba^2+^ ions (1 mM) and Tetrodotoxin (TTX; 0.001 mM) were added to prevent activation of K^+^ currents and Na^+^ spikes. Patch pipettes (2.5–3.5 MΩ, 1.5 OD × 1.17 × 100 L mm, Harvard apparatus, Cambridge, UK) were filled with intracellular solution (containing in mM): 140 KMeSO_4_, 10 HEPES, 10 KCl, 10 phosphocreatine, 4 MgATP, 0.2 NaGTP, 0.1 EGTA, pH 7.3, 305 mOsm. NaGTP was freshly added daily from 100-fold concentrated stock solutions.

### Electroretinography (ERG)

ERGs were performed according to procedures described previously (Tanimoto et al., 2009). Mice were dark-adapted overnight, and then anesthetized by IP injection of ketamine (66.7 mg/kg) and xylazine (11.7 mg/kg). Body temperature was maintained at 37°C using a heating pad. Mouse pupils were dilated by topical administration of Mydriaticum, and custom-made gold wired electrodes were placed on the corneas. ERG responses were recorded simultaneously from both eyes under dark-adapted conditions (without any background illumination). Responses were recorded using white flashes of 0.1, 1, 10, 100, 1,000, 10,000 and 25,000 mcd^*^s/m^2^.

### Scanning-laser ophthalmoscopy (SLO)

SLO imaging was performed with a HRA 1 system (Heidelberg Engineering, Heidelberg, Germany). The HRA 1 features two lasers in the short (visible) wavelength range (488 and 514 nm), and in the long (infrared) wavelength range (795 and 830 nm). The 488 and 795 nm lasers are used for fluorescein (FLA) and indocyanine green (ICG) angiography, respectively. Mice were measured according to previously described procedures (Seeliger et al., 2005). In short, mice were anesthetized by subcutaneous injection of ketamine (66.7 mg/kg) and xylazine (11.7 mg/kg), and their pupils dilated with tropicamide eye drops (Mydriaticum Stulln, Pharma Stulln, Stulln, Germany). A custom-made contact lens was used to avoid dehydration of the cornea. FLA was performed using a s.c. injection of 75 mg/kg body weight fluorescein-Na (University pharmacy, University of Tuebingen, Germany), and ICGA following a s.c. injection of 50 mg/kg body weight ICG (ICG-Pulsion, Pulsion Medical Systems AG, Munich, Germany).

### Circadian experiments

Experiments were done according to (Jud et al., 2005). Eight wild type and 10 HCN3^−/−^ animals were entrained to a 12 h light 12 h dark cycle in a new cage that contained the running wheel (LD12/12) with lights switched on at 7 a.m. for 7–15 days before they were released to constant darkness (DD) or constant light (LL) for 18 days. Activity was assessed with a running-wheel and evaluated using ClockLab (Actimetrics). Activity records were double plotted in threshold format for 6-min bins. Period length was assessed by χ^2^ periodogram analysis for days 4–10 in DD or LL. To determine light induced phase shifts, an Aschoff Type I protocol was used (Aschoff, 1977). Animals were allowed to stabilize their free-running rhythm (at least 14 days) prior to the light pulse. The circadian time (CT) at the beginning of the light pulse was calculated for every mouse individually. Fifteen minutes light pulses (400 lux, white light) were applied at CT10 (control), CT14 and CT22. Light applied at CT10 corresponds to light perceived during the subjective day and did not provoke a phase shift of behavioral activity. Light pulses at CT14 and CT22 fall at early or late subjective night and lead to phase delays or advances, respectively. Between the different light pulses, mice were allowed to stabilize their circadian oscillator for 3 weeks. Phase shifts were determined by fitting a regression line through onsets of activity before the light pulse and a second regression line through onsets of activity after the light pulse. The distance between the two regression lines on the day following the light pulse determined the amount of phase shift. Further, we tested how the two genotypes behaved in a jet-lag experiment. Animals were kept in a 12:12 LD cycle for at least 10 days. Then the LD cycle was delayed by 6 h (forwards or backwards) and the number of days for adaptation to the new LD cycle was measured.

### Behavioral experiments

For behavioral experiments HCN3^−/−^ animals and aged matched control wild type littermates were used. Animals were single housed at least 1 week prior to the experiments, with food and water *ad-libitum* under an inverse 12 h light/dark cycle, where lights were switched off at 7 a.m. All behavioral experiments were performed during the active phase of the mice between 9 a.m. and 5 p.m. and analyzed blindly to the genotype of the animals. The (i) Morris water maze, (ii) dark-light-transition test, and (iii) rotarod experiments were performed using the same 18 wild type and 13 knockout animals, in the given order. Furthermore, the same 10 wild type and 10 knockout animals were tested in the (i) open field and later in the (ii) fear conditioning paradigm. For all other experiments separate cohorts of animals were used as indicated.

### Morris water maze

18 wild type and 13 knockout animals aged 10–12 weeks were tested in the morris water maze using a protocol adapted from Morris (1984) and Fritz et al. (2017). The task was performed in a circular tank (diameter: 150 cm, height: 68 cm, filling level: 15 cm) filled with water (24–26°C) to 20 cm and surrounded by visual cues. The water was made opaque by adding sufficient amount of milk. During the training period, an escape platform (12 × 12 cm) was placed in the middle of a designated target quadrant 1 cm below the water surface. Mice were dropped into the basin at pseudorandomized locations. On the first 3 days (training) mice were trained to find the platform by six 120 s trials. A trial ended either when an animal rested on the hidden platform for 10 or 120 s had elapsed (in that case, mice were guided to the platform). On day 4 the reverse hidden platform learning task was started by moving the platform to the diagonally opposite quadrant. Mice were tested in 6 runs on two consecutive days. All movement was videotaped and the tracking system Ethovision 2.2 (Noldus) was used. Parameters analyzed off-line by means of the software WinTrack were: time/distance until platform is reached, time in trained quadrant, time in other quadrants, thigmotaxis, floating, and others.

### Dark-light-transition test

18 wild type and 13 knockout animals were tested for anxiety in the dark-light-transition test (adapted from Crawley and Goodwin, 1980). This test was conducted in a square arena (20 × 45 cm) in which a black plastic box (20 × 15 cm) was used to separate adjoining dark and well-lit areas (411 lux). Mice were placed in the center of the bright area and allowed to explore the arena for 10 min. Movements were videotaped and the tracking system used was Ethovision 2.2 (Noldus). The arena was cleaned with 70% alcohol between tests. Parameters analyzed were: latency to enter dark area, time in light area, time in dark box, number of transitions between light and dark compartment, rearing, and grooming.

### Rotarod

18 wild type and 13 knockout animals were tested in the rotarod using an adaptation of a protocol previously described (Nolan et al., 2003). To measure motor coordination the accelerating rotarod apparatus (Ugo Basile) was used. During the training period, which took place on 2 days with 4 runs each day, mice were placed on the rotarod (3 cm diameter) accelerating to 40 rpm. The maximum observation time was 5 min. During testing on the third day animals received three consecutive 2 min trials at constant speeds of 37.5, 28.5, 21.5, and 11.5 rpm. The latency to fall (or pegging onto the rotating latch) was measured.

### Elevated plus maze (EPM)

Male adult (13–20 weeks) wild type (*n* = 10) and knockout (*n* = 9) animals were tested in the EPM using a protocol described previously (Pellow and File, 1986) adapted to assess mouse behavior. The apparatus consisted of two opposite open arms, (20 × 5 cm) and two opposite closed arms (20 × 5 × 14 cm) connected to a central platform (5 × 5 cm) to form a cross. The maze was elevated 50 cm from the floor. Illumination was adjusted to 50 lux. The animals were placed at the center of the maze with the nose in the direction of one of the closed arms, and observed for 5 min, according to the following parameters: number of entries in the open and closed arms, and time of permanence in each of them (i.e., the time spent by the animal in the open and closed arms). An entry was defined as all four paws having crossed the imaginary line between an arm and the central area. On removal of each mouse, the maze floor was carefully wiped with 70% EtOH. The behavior of all animals was recorded using a video camera and analyzed offline using the TSE maze systems software.

### Marble-burying test

Based on the protocol previously published by Deacon (2006), 10 wild type and 10 knockout animals (17–19 weeks old) were tested in their home cage (35.5 × 20.7 cm) filled with fresh bedding to 5 cm. 12 glass marbles (1.4 cm diameter) were arranged in 3 × 4 equidistant rows. After 30 min under low-light conditions, the number of marbles that were at least covered for 2/3 was determined.

### Forced swimming test (FST)

Using an adaptation of the FST protocol described in Castagné et al. (2011), 20 wild type and 20 knockout animals were tested in standard 5 l glass beakers (height 25 cm; Schott) filled with water (26 ± 0.3°C) to 10 cm, so that the animals could neither touch the bottom with their tails, nor could escape from the top. Two beakers were separated by an opaque screen, so that two animals (WT and litter-matched HCN3^−/−^) could be tested at the same time. Between sessions, the beakers were cleaned thoroughly and fresh water was added. At the start of each test, an animal was gently picked up by its tail from the home cage and rapidly placed into the middle of the beaker. At the time the animal was placed in the water, the recording time was started and the duration of each standard FST was set to 6 min. After the test, the animal was removed from the water, dried with a towel and put into a warm cage (temperature of bedding 31–33°C) for 15 min before returning to their home cage. The entire session was videotaped (Logitech Quick Cam) and analyzed off-line using a software (Winrat.exe version2.3.1) by an experienced experimenter. Mice were tested again 24 h later.

### Open field test

To assess locomotor activity, wild type (*n* = 10) and HCN3^−/−^ (*n* = 10) animals at an age of 6–9 weeks were tested in the open field under low light conditions, using a protocol adapted from Leitinger et al. (1994). The open field chamber used was a TSE conditioning chamber (25 × 25 × 25 cm) covered with acoustic foam (Conrad electronics). The test was initiated by placing mice in the middle of the open field and allowing them to move freely for 10 min, while being tracked by light barriers. The chamber was cleaned with 70% EtOH between tests. Testing was repeated 24 h later. Parameters analyzed were distance moved, velocity, and time being active (movement speed > 3 cm/s).

### Fear conditioning

Male wild type (*n* = 10) and HCN3^−/−^ (*n* = 10) animals at an age of 6–9 weeks were used. The setup for the fear conditioning test has been described and displayed in detail before (Kamprath and Wotjak, 2004). Briefly, the experiments were performed in two contexts: (1) the shock chamber was a cubic-shaped box with a metal grid for shock application (conditioning chamber, TSE) and (2) the neutral test context, which was cylindrically shaped (25 cm diameter) and made of transparent acrylic glass, with wood shavings as bedding. The two contexts were cleaned thoroughly after each trial with differently smelling detergents, and bedding was changed. For conditioning (d0), mice were placed into the shock chamber. After 180 s a tone was presented (9 kHz, 80 dB, 30 s), which co-terminated with a single scrambled electric foot shock (2 s, 0.7 mA). Animals remained in the shock chamber for another 30 s before they were returned to their home cages. On day 1 and 2 after conditioning, mice were exposed either to the tone (180 s) in the neutral test context (d1) or to the conditioning context (180 s). One week later the exposure to the tone in the neutral or the conditioning context was repeated. The behavioral performance was videotaped by a video camera (Eneo VK-1316s, TSE), and animals' behavior was analyzed off-line by a trained observer who was unaware of the genotypes. Freezing behavior was defined as immobility except for respiration movements and served as a measure of fear memory.

### Statistical analysis

Data were analyzed in GraphPad Prism (version 7.01) using χ^2^, unpaired *t*-test or two-factor analysis of variance for repeated measures and the *post-hoc* Bonferroni test for multiple comparisons if appropriate. Statistical significance was accepted if *P* ≤ 0.05.

## Results

For the present study we used the previously published knockout mouse line, HCN3^−/−^, in which HCN3 is globally deleted (Fenske et al., 2011). HCN3-deficient mice were born at the expected Mendelian ratio, were fertile, and showed no immediately visible physical abnormalities. The body weights were similar in wild type and HCN3^−/−^ mice (wild type: 29.64 ± 1.29 g; HCN3^−/−^: 31.27 ± 1.31 g; *t* = 0.8866, *p* > 0.05) 12 weeks after birth. Furthermore, organ specific laboratory parameters including plasma cholesterol, lipoprotein panel, liver function tests (total plasma proteins, transaminases alkaline phosphatase), exocrine pancreatic function (amylase and lipase) and blood glucose levels were indistinguishable between wild type and HCN3^−/−^ animals under baseline conditions (Table 1). Moreover, plasma electrolyte concentrations were similar in both group of mice (see Table 1) with only mildly decreased Ca^2+^ concentrations and urea levels in HCN3^−/−^ mice as compared to wild type mice. Creatinine and uric acid were normal.

**Table 1:** Blood plasma parameters.

**Parameter**	**WT**	**HCN3^−/−^**	**Unit**	**Method**
Na^+^	158 ± 0.51	156.5 ± 1.47	mmol/l	Ion selective electrode
K^+^	3.86 ± 0.12	3.73 ± 0.08	mmol/l	Ion selective electrode
Ca^2+^	2.07 ± 0.03	1.96 ± 0.03^*^	mmol/l	Photometric Color Test
Cl^−^	114.58 ± 0.62	114.25 ± 1.10	mmol/l	Ion selective electrode
${\text{PO}}_{4}^{3-}$	1.71 ± 0.07	1.73 ± 0.08	mmol/l	Photometric UV Test
Creatinine	0.32 ± 0.01	0.32 ± 0.0036	mg/dl	Kinetic Color Test
Urea	46.62 ± 1.78	38.95 ± 1.67^**^	mg/dl	Kinetic UV Test
Uric acid	0.47 ± 0.18	0.27 ± 0.16	mg/dl	Enzymatic Color Test
**LIPID METABOLISM**				
Cholesterol	75.92 ± 4.04	76.60 ± 4.69	mg/dl	Enzymatic Color Test
Triglycerides	117.26 ± 12.12	111.59 ± 9.13	mg/dl	Enzymatic Color Test
LDL	15.93 ± 0.86	16.45 ± 1.14	mg/dl	Enzymatic Color Test
HDL	50.67 ± 2.84	49.93 ± 2.98	mg/dl	Enzymatic Color Test
**LIVER FUNCTION**				
Total protein	5.13 ± 0.1	4.85 ± 0.12	g/dl	Photometric Color Test
Alanine transaminase	22.35 ± 2.04	21.38 ± 2.52	U/l	Kinetic UV Test
Aspartate transaminase	62.47 ± 6.93	54.63 ± 5.94	U/l	Kinetic UV Test
Alkaline phosphatase	126.94 ± 8.82	125.25 ± 13.40	U/l	Kinetic Color Test
Ferritin	47.29 ± 4.62	45.23 ± 4.74	μg/l	Immuno-turbidimetric Test
Transferrin	147.41 ± 2.27	145.23 ± 1.55	mg/dl	Immuno-Turbidimetric Test
**PANCREATIC FUNCTION**				
Glucose	155.35 ± 6.41	162.21 ± 6.48	mg/dl	Enzymatic UV Test
Alpha-amylase	2310.00 ± 72.97	2393.25 ± 58.52	U/l	Kinetic Color Test
Lipase	53.77 ± 2.45	51.77 ± 2.07	U/l	Kinetic Color Test
Creatine kinase	578.12 ± 42.69	497.38 ± 46.55	U/l	Kinetic UV Test

Parameters were assessed from blood plasma of 10 wild type and 11 HCN3^−/−^ animals.

* p < 0.05,

** *p < 0.01*.

In order to detect HCN3 protein we previously generated a HCN3 antibody raised against the C-terminus of HCN3 as described in Mistrik et al. (2005). In the absence of preabsorption against HCN3^−/−^ lysates unspecific signal including nuclear staining was present (not shown). Nuclear staining of HCN3 using non preabsorbed antibody seems to be present in Ying et al. (2011) and Peng et al. (2017). To reduce unspecific binding we therefore only used HCN3 antibody preabsorbed against HCN3^−/−^ brain lysates throughout this study as described in Fenske et al. (2011). We first confirmed the specificity of the HCN3 antibody (Supplementary Figure 1) in the brain. A side by side comparison of cryosections prepared from wild type and HCN3^−/−^ brains revealed the presence of HCN3 in wild type and the absence of HCN3 in the HCN3^−/−^ brain, indicating that the HCN3 antibody specifically detects HCN3 protein. There is however unspecific staining in vascular structures including the choreoid plexus. Immunohistochemical (IHC) experiments and Western blots (For examples see Supplementary Figures 1, 2, respectively) revealed that HCN3 is broadly expressed in the CNS, in particular in the cortex, amygdala, hippocampus, the hypothalamus, pituitary gland, thalamus, brainstem, olfactory system, retina, and the intergeniculate leaflet (IGL) of the circadian system (Table 2). Inspired by the distinct expression pattern of HCN3 in the CNS we characterized the expression and function of these channels in the circadian system and their role for behavior in more detail.

**Table 2:** Expression of HCN3 channels.

**Name of the structure**	**Expression level**
*Hypothalamus*^a^	
Hypophysis	++
Nucleus dorsomedialis	++
Nucleus hypothalamicus anterior	++
Nucleus lateralis	++
Nucleus paraventricularis	++
Nucleus preopticus medialis	++
Nucleus supraopticus	++
Suprachiasmatic nucleus	–
*Thalamus*	
Corpus geniculatum mediale	–
Intergeniculate leaflet	++
Lateral geniculate nucleus dorsal part	–
Lateral geniculate nucleus ventral part	++
Nucleus habenularis laterlais	++
Nucleus habenularis medialis	++
Nucleus laterodorsalis	++
Nucleus paraventricularis Thalamicus	++
Nucleus reticularis	++
*Brainstem*	
Locus coeruleus	++
Nucleus parabrachialis lateralis	++
Nucleus parabrachialis medialis	++
Nucleus subparabrachialis	++
Nucleus tegmentalis, laterodorsal part	++
Periaqueductal gray	++
Raphe nuclei dorsal part	++
Raphe nuclei medial part	++
Substantia nigra pars compacta	++
*Limbic system*	
Basolateral Amygdala	++
Hippocampus, str. Lacunosum moleculare	+
*Main olfactory bulb*^a^	
Glomerular layer	++
Internal plexiform layer	++
*Telencephalon*	
Cortex^a^	+
Nucleus basalis	++
Striatum	++
*Cerebellum*^a^	+
*Retina*^a^	
Inner plexiform layer	++
Outer plexiform layer	+
*Outside CNS*	
Heart	+

Expression data was obtained using Immunohistochemistry.

a *Presence of HCN3 protein was confirmed with Western blots*.

### Role of HCN3 channels in the circadian system

Circadian rhythms, including timing of wakefulness and sleep, are mainly controlled by the suprachiasmatic nucleus of the hypothalamus (SCN) which acts as a master clock controlling circadian behavior (Figure 1; Albrecht et al., 2012). This clock is synchronized (or entrained) to day-night rhythm by the activity of brain regions, which provide information about light (photic entrainment by the retina), arousal or availability of food (both non-photic entrainment). Among these regions, the intergeniculate leaflet (IGL) has been suggested to be critical for the integration and for transmission of these photic and non-photic entrainment cues to the SCN (Figure 1; Delogu et al., 2012). The IGL is located upstream of the SCN within the thalamus forming a leaflet in between the medial and lateral geniculate nuclei. The dominant entrainment cue for the circadian system is light (photic entrainment). Light input signal for the circadian system is detected by the retina and transferred via the retinohypothalamic tract (RHT) to the SCN and the IGL. Given the central role of the retina, the SCN, and the IGL for the control of circadian rhythm we next analyzed the expression and the functional role of HCN3 channels in these regions.

**Figure 2:**
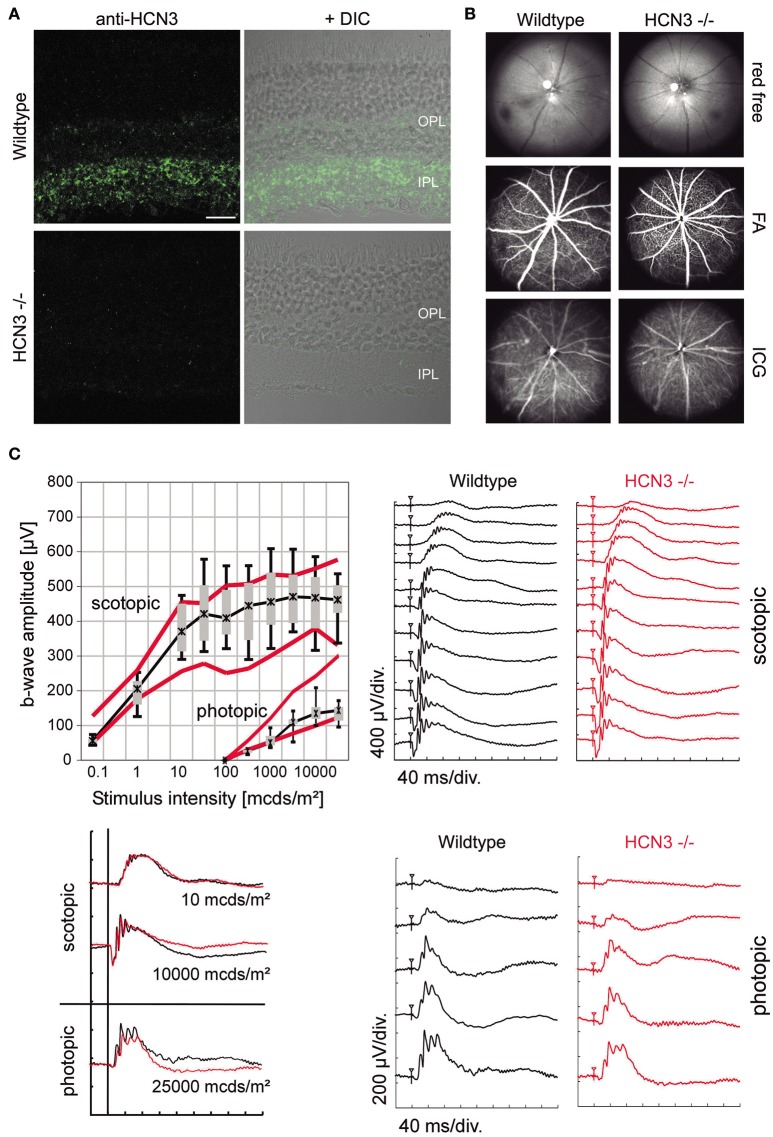
Characterization of retinal structure and function in HCN3^−/−^ mice. **(A)** Expression of HCN3 in the retina. Wild type mice express HCN3 (green) in the inner plexiform layer (IPL) and the outer plexiform layer (OPL). The immunolabeling is absent in HCN3^−/−^ mice. For visualization of the retinal layers, confocal fluorescent images are overlaid with DIC images of the same area. Scale bar, 20 μm. **(B,C)** Examination of retinal structure and function *in vivo* [wild type control mice (*n* = 5) and HCN3^−/−^ (*n* = 5), age 3 months]. **(B)** Retinal imaging and angiography. Mice were examined with native SLO (red-free, 514 nm), fluorescein angiography (488 nm, barrier filter at 500 nm), and indocyanine green (ICG) angiography (795 nm, barrier filter at 800 nm). HCN3^−/−^ mice show no signs of neuroretinal or vascular alterations. **(C)** Retinal function assessment via ERG. Top right, Representative scotopic ERG intensity series (dark-adapted conditions). Bottom right, Representative photopic ERG intensity series (light-adapted conditions). Top left, Quantitative scotopic and photopic b-wave amplitude data from HCN3^−/−^ mice (box plot). Boxes: 25–75% quartile range, whiskers: 5 and 95% quantiles, asterisks: median. As a control, red bolded lines indicate the 25 and 75% quartile of the wild type group. bottom left, Overlay of representative ERG recordings under scotopic (0.01 and 10 cd^*^s/m^2^) and photopic (25 cd^*^s/m^2^) conditions. Wild type black traces, HCN3^−/−^ red traces.

IHC experiments revealed distinct immune labeling for HCN3 in the retina (Table 2, Figure 2A). HCN3 signal is mainly found in the inner and to a lesser extent in the outer plexiform layer, which harbor the retinal synapses between bipolar and ganglion cells, and between photoreceptors and bipolar cells, respectively. Retinal *in vivo* imaging and fluorescein angiography via scanning-laser ophthalmoscopy (SLO) did not provide any evidence for morphological changes to neuroretinal or vascular structures in HCN3^−/−^ animals (Figure 2B). Retinal function, analyzed via electroretinography (ERG) under scotopic (dark-adapted) and photopic (light-adapted) conditions, was normal in HCN3^−/−^ mice (Figure 2C). In particular, amplitude and shape of the b-wave, which reflects directly the activity of bipolar cells in the inner retina and indirectly activity of photoreceptors and successful synaptic transmission at the first synapse (Tanimoto et al., 2009), were normal (Figure 2C, left). Overall, results obtained from ERG studies indicate no obvious role of HCN3 channels for processing of electrical signals in the retina.

**Figure 3:**
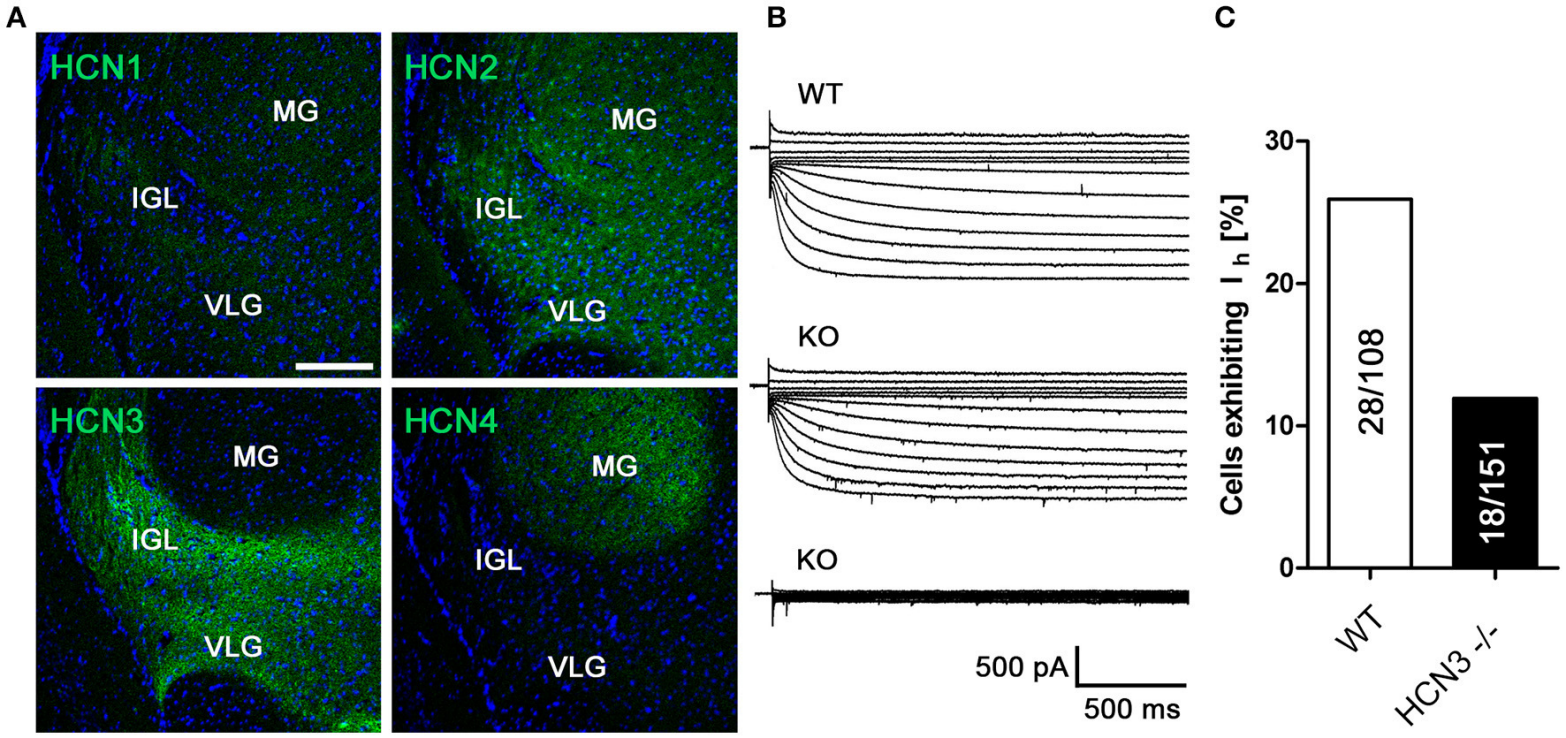
HCN3 channels are expressed in the intergeniculate leaflet and contribute to I_h_. **(A)** Expression of HCN channel subtypes in the intergeniculate leaflet (IGL), medial geniculate nucleus (MG) and ventral geniculate nucleus (VLG); Scale bar, 200 μm. **(B)** Family of current traces for I_h_ recordings in acute slice preparations containing the IGL. Upper panel: recordings from WT preparations. In slices prepared from HCN3^−/−^ mice, two populations of cells were present, one containing I_h_ (middle panel), which is most likely carried by HCN2, and one without I_h_ (lower panel). **(C)** Number of tested IGL neurons exhibiting the I_h_ current in relation to the number of tested cells given in %.

Given the central role of the SCN for the regulation of circadian rhythm, we next performed IHC experiments in this specific nucleus and in neighboring hypothalamic nuclei. Interestingly, the immunosignal for HCN3 in the SCN was very weak compared to the surrounding cell populations (Supplementary Figure 3). HCN3 is abundantly expressed in other nuclei of the hypothalamus (Nucl. paraventricularis, Nucl. dorsomedialis, Nucl. lateralis, Nucl. hypothalamicus anterior and Nucl. preopticus medialis; Table 2; Supplementary Figure 4), which are not related to circadian function. However, we did not find any evidence for specific functional defects of HCN3^−/−^ mice including corticosterone, fluid and electrolyte homeostasis controlled by these nuclei (Supplementary Figure 4). Remarkably, and in line with previous reports (Ying et al., 2011), we were able to confirm pronounced expression of HCN3 in the IGL (Figures 3A,B; Table 2), which was absent in HCN3^−/−^ animals (Supplementary Figure 1). In addition to HCN3, HCN2 channels are expressed in the IGL, while HCN1 and HCN4 are hardly detectable. I_h_ recordings in wild type and HCN3^−/−^ mice confirm that HCN3 channels contribute to I_h_ in the IGL (Figure 3B). Furthermore, in WT 28/108 neurons in the IGL (25%) displayed I_h_ while in KO significantly less cells, 18/151 (10%), displayed this current (χ^2^ = 8.456; *p* < 0.01) (Figure 3C). This indicates that different populations of neurons are present in the IGL, one population in which I_h_ is entirely conferred by HCN3 and a second population which express a HCN isoform that is not affected by the knockout of HCN3, most likely HCN2.

**Figure 4:**
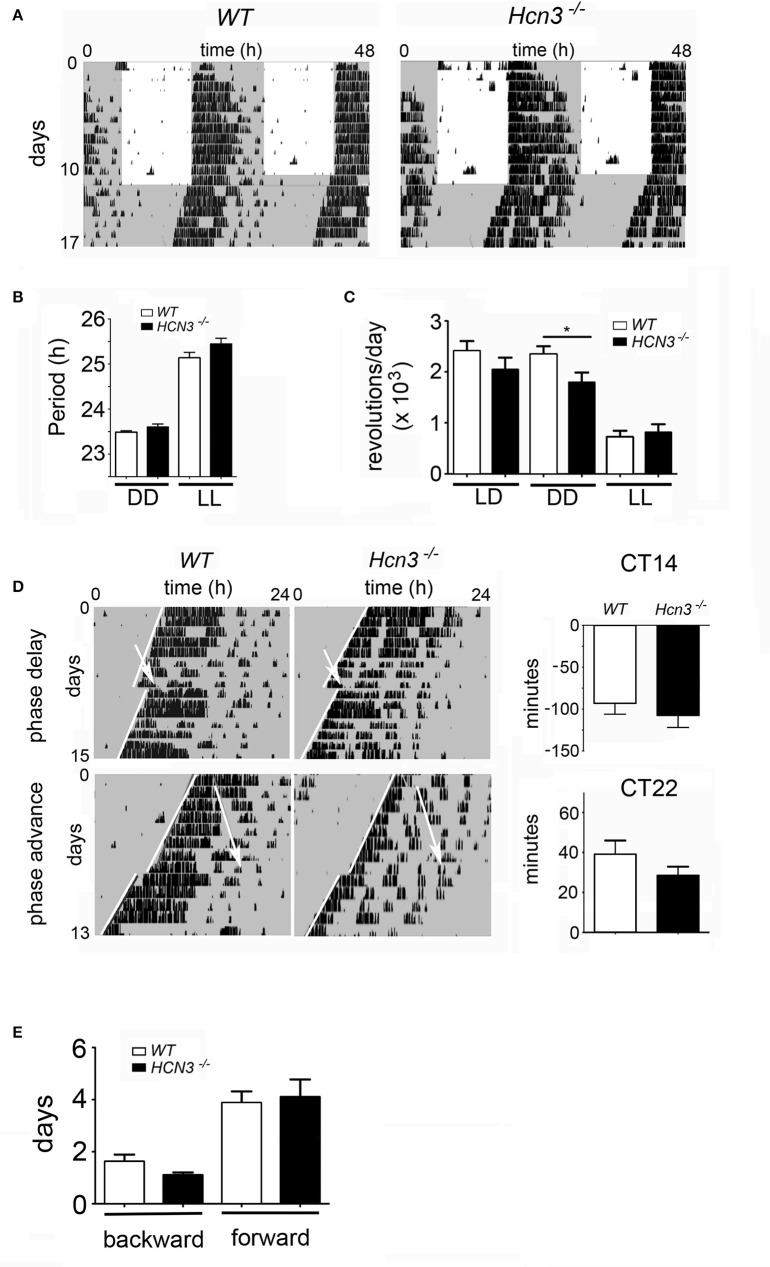
Circadian clock parameters of HCN3^−/−^ mice. **(A)** Wheel-running activity of wild type (left) and HCN3^−/−^ (right) mice. Gray areas indicate darkness and white areas light. The actograms are double plotted and display two consecutive days per line. **(B)** Quantification of the period under constant darkness (DD) and constant light (LL) of wild type (black bars) and HCN3^−/−^ (white bars) mice. Under DD conditions the periods were 23.49 ± 0.03 h (*n* = 7) and 23.6 ± 0.07 h (*n* = 9) and under LL conditions they were 25.14 ± 0.12 h (*n* = 7) and 25.45 ± 0.13 h (*n* = 9), respectively. **(C)** Total activity in the two genotypes under light dark (LD), DD, and LL conditions. Wheel revolutions (rev) per day were counted and were as follows: LD conditions, WT 24091 ± 1960 rev (*n* = 8), HCN3^−/−^ 20439 ± 2357 rev (*n* = 10), DD conditions, WT 23482 ± 1555 rev (*n* = 8), HCN3^−/−^ 17925 ± 1945 rev (*n* = 10), LL conditions, WT 7200 ± 1241 rev (*n* = 8), HCN3^−/−^ 8118 ± 1613 rev (*n* = 10). **(D)** Clock-resetting in response to a light pulse at CT14 (phase delay) and CT22 (phase advance). White arrows indicate the time of application of the light pulse. White lines delineate activity onset, before (top) and after (bottom) the light pulse. The phase delays were: WT −93.29 ± 12.88 min (*n* = 7) and HCN3^−/−^ −107.8 ± 14.24 min (*n* = 10). The phase advances were: WT 39 ± 7.01 min (*n* = 5) and HCN3^−/−^ 28.44 ± 4.45 min (*n* = 9). **(E)** Adaptation time to a jet-lag delaying the onset of light by 6 h (backward shift) or advancing the onset of light by 6 h (forward shift). The adaptation times were: backward, WT 1.63 ± 0.26 days (*n* = 8) and HCN3^−/−^ 1.1 ± 0.1 days (*n* = 10), forward, WT 3.88 ± 0.44 days (*n* = 8) and HCN3^−/−^ 4.1 ± 0.67 days (*n* = 10). All values are Mean ± SEM, unpaired two-tailed *t*-test, ^*^p < 0.05.

To evaluate whether HCN3 channels in the IGL play a substantial role in the regulation of the circadian clock, we characterized the daily activity in wild type and HCN3^−/−^ mice (Figure 4). Animals were entrained to a 12 h light/dark (LD) cycle (12:12 LD) for 15 days and then released into constant darkness (DD) for 18 days (Figure 4A). The two groups displayed similar free-running activity rhythms (period length) under DD (WT: 23.49 ± 0.03 h; KO: 23.6 ± 0.07 h; *t* = 1.307, *p* > 0.05) as well as under LL (24 h light; WT: 25.14 ± 0.12 h; KO: 25.45 ± 0.13 h; *t* = 1.705, *p* > 0.05) conditions (Figure 4B). Interestingly, the precision of activity onset was similar in the two genotypes under LD (Figure 4A) as well as under DD (Figure 4D) conditions. Furthermore, the two groups did not differ significantly in total activity under LD (WT: 24,091 ± 1,960 rev; KO: 20,439 ± 2,357 rev; *t* = 1.152, *p* > 0.05) and LL (WT: 7,200 ± 1,241 rev; KO: 8,118 ± 1613 rev; *t* = 0.4325, *p* > 0.05) conditions (Figure 4C), however under DD conditions the HCN3^−/−^ animals displayed significantly less activity (17925 ± 1945 wheel-revolutions, *n* = 10) compared to their controls (23482 ± 1555 wheel-revolutions, *n* = 8; *t* = 2.148, *p* < 0.05). These results suggest that HCN3 channels can affect the total amount of activity in the absence of a light cue, but do not play a major role in the regulation of circadian timing under constant lighting conditions.

Because it is well-known that cAMP-dependent signaling and neuronal excitation are both involved in the light-dependent entrainment process of the circadian clock, we tested the potential role of HCN3 channels in the regulation of the response of the circadian clock to a nocturnal light stimulus. We analyzed the clock resetting behavior of HCN3^−/−^ mice and compared it to wild type animals. Mice of both genotypes were held in DD for at least 14 days and then exposed to a 20-min light pulse. Subsequently, the animals were kept for an additional 16 days in DD, and their phase-shifts were assessed. As expected, both genotypes, control and HCN3^−/−^ mice, did not display phase-shifts when subjected to light at CT10 (data not shown), because this time point corresponds to subjective day (CT0-12), where the clock is insensitive to light. Exposure to a light pulse in the early subjective night (CT14), evoked normal phase delays in both control and HCN3^−/−^ mice (−93.29 ± 12.88 min, −107.8 ± 14.24 min, respectively; *t* = 0.7181, *p* > 0.05; Figure 4D, top panels). Likewise, application of a light pulse in the late subjective night (CT22) evoked a phase advance in both genotypes (WT: 39 ± 7.01; KO: 28.44 ± 4.45 min; *t* = 1.336, *p* > 0.05; Figure 4D bottom panels). There was no difference in the amount of phase delays and phase advances between HCN3^−/−^ mice and wild type control animals (Figure 4D). Overall, these results suggest that the HCN3 channel does not play a major role in the light-induced resetting mechanism of the circadian clock.

In a next step, we tested how the two genotypes behaved in a jet-lag experiment. Animals were kept in a 12:12 LD cycle for at least 10 days. Then the LD cycle was delayed by 6 h and the number of days for adaptation to the new LD cycle was measured. For both genotypes this backward shift of the clock was similar and took <2 days (WT: 1.63 ± 0.26 days; KO: 1.1 ± 0.1 days; *t* = 2.065, *p* > 0.05; Figure 4E). Advancing the LD cycle by 6 h took the animals of both genotypes around 4 days (WT: 3.88 ± 0.44 days; KO: 4.1 ± 0.67 days; *t* = 0.2592, *p* > 0.05; Figure 4E). These observations indicate that the HCN3 channel does not play an important role in the speed of adaptation in the behavior of mice to a new LD cycle.

### Role of HCN3 channels for the control of behavior

We performed a set of behavioral tasks for motor learning, spatial learning, and memory, exploratory behavior as well as emotionality such as depression-related behavior and stereotypic perseverative behavior.

**Figure 5:**
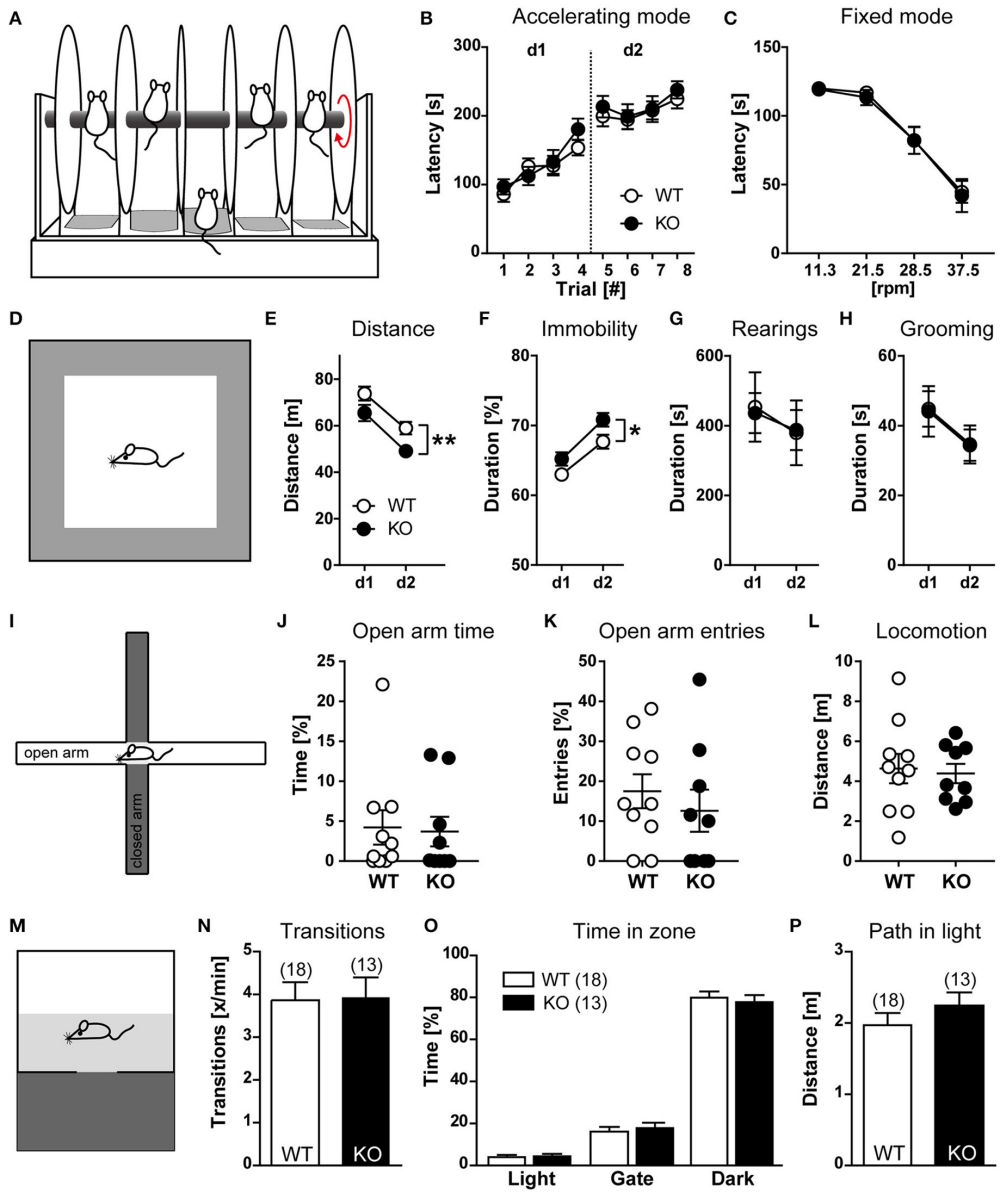
Behavioral characterization of HCN3^−/−^ mice (part 1). **(A)** Schematic of the rotarod test. Mice were placed on drums rotating at an accelerating rotation speed **(B)** or at the rotation speed indicated **(C)**. The time at which an animal fell from the drum was measured. The increased latency from trial 1 to trial 8 indicates motor learning behavior **(B)**. **(D)** Schematic of the open field test. **(E–H)** Open field performance on two consecutive days. Monitoring of general locomotor activity in a novel environment over 10 min. The distance traveled **(E)**, relative time of immobility **(F)**, rearings **(G)**, and grooming **(H)** are indicated. **(I–L)** Elevated plus maze. Schematic of the maze **(I)**. Analysis of time spent in open arms **(J)**, entries into open arms **(K)**, and total locomotion **(L)** revealed no genotype differences. **(M)** Schematic of the light-dark box. **(N–P)** In the light-dark box no genotype differences were evident since all animals showed the same number of transitions **(N)**, avoided the brightly illuminated part of the chamber **(O)**, and traveled a similar distance in the bright area **(P)**. All values are Mean ± SEM, ^*^p < 0.05, ^**^p < 0.01.

To evaluate whether HCN3 channels are involved in the control of motor function, wild type and HCN3^−/−^ mice were trained for 2 days with 4 trials per day to balance on a rod accelerating from 4 to 40 rpm over 5 min, and the latency at which mice fell from the accelerating rod was recorded (Figures 5A,B). Using this rotarod protocol, motor coordination, skilled movements and motor learning can be evaluated. Both, WT and HCN3^−/−^ mice showed motor learning on the rotarod (accelerating mode) as indicated by the increase in fall-off latencies [Trial: *F*_(7, 203)_ = 40.22, *p* < 0.0001; 2-way ANOVA for repeated measures] with no genotype differences [GT: *F*_(1, 29)_ = 0.3251, *p* > 0.05; GT × Trial: *F*_(7, 203)_ = 0.5523, *p* > 0.05; Figure 5B]. On day 3, mice were placed on a rotarod of constant speed (fixed mode) at 37.5, 28.5, 21.5, and 11.3 rpm, and the latencies at which mice fell from the rod were determined. In the fixed mode, fall-down latencies were clearly dependent on the speed of the rod [rpm: *F*_(3, 87)_ = 70.73, *p* < 0.0001; 2-way ANOVA for repeated measures] with no genotype differences [GT: *F*_(1, 29)_ = 0.054, *p* > 0.05; GT × rpm: *F*_(3, 87)_ = 0.042, *p* > 0.05; Figure 5C]. These findings indicate that HCN3 is dispensable for motor learning and motor coordination.

Exploration-based behavior was tested using the open field test (Figure 5D). For this test, mice were exposed to an Open Field for 10 min on two consecutive days. On the second day of testing, KO animals showed reduced horizontal exploration [GT: *F*_(1, 18)_ = 11.97, *p* < 0.01; 2-way ANOVA for repeated measures; Figure 5E], which declined to the same extent in both genotypes [Day: *F*_(1, 18)_ = 26.0, *p* < 0.0001; GT × Day: *F*_(1, 18)_ = 0.0666, *p* > 0.05]. The main effect of genotype can be ascribed to a general increase in immobility in KO mice [GT: *F*_(1, 18)_ = 8.034, *p* < 0.05; Figure 5F], which was also observed in circadian experiments under constant darkness. In contrast, vertical exploration [rearing; GT: *F*_(1, 18)_ = 0.0023, *p* > 0.05; Figure 5G] and grooming [GT: *F*_(1, 18)_ = 0.0044, *p* > 0.05; Figure 5H] were unaffected.

In the elevated plus maze (Figure 5I), another independent test for exploration-based behavior, both, WT and HCN3^−/−^ mice avoided exploration of the aversive open arms to the same extent (Figures 5J,K) and showed comparable levels of overall locomotor activity (Figure 5L). These results indicate that explorative and anxiety-like behavior are similar in HCN3^−/−^ and wild type mice.

In line with this interpretation in the dark-light test (Figure 5M), another test for exploration-based anxiety, HCN3^−/−^ and wild type mice behaved similar. The number of transitions between illuminated and dark chambers was the same in wild type and HCN3^−/−^ mice (Figure 5N). Furthermore, both groups of mice strongly avoided the brightly illuminated chamber in favor of the dark compartment (Figure 5O). There was no difference in the number of entries into (wild type: 3.87 ± 0.42; HCN3^−/−^: 3.93 ± 0.47; *t* = 0.1024, *p* > 0.05) and the time spent in the dark compartment (wild type: 79.9 ± 2.9%; HCN3^−/−^: 77.8 ± 3.1%, *p* > 0.05) for both group of mice. Finally, WT and HCN3^−/−^ animals showed similar levels of activity in the light area as evident from the total distance mice traveled there (Figure 5P; wild type: 1.97 ± 0.17; HCN3^−/−^: 2.24 ± 0.19, *t* = 1.050, *p* > 0.05).

**Figure 6:**
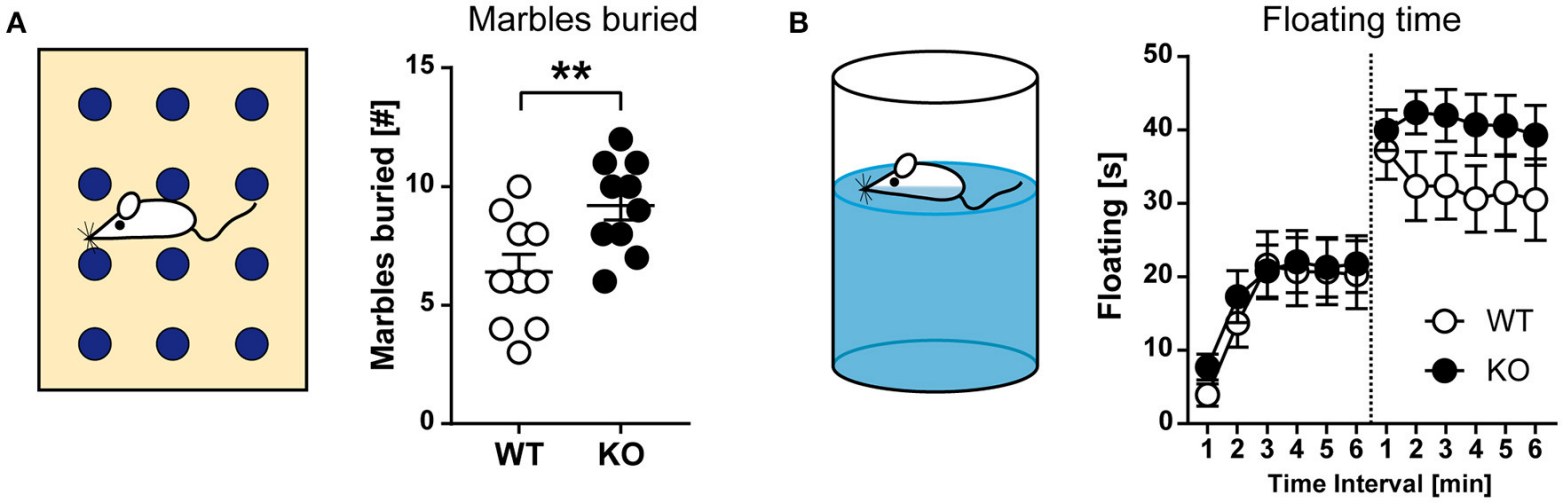
Behavioral characterization of HCN3^−/−^ mice (part 2). **(A)** Marble burying test. Schematic of the testing environment (left), where mice exposed to a cage containing 12 identical glass marbles. HCN3^−/−^ mice buried more marbles as compared to wild type littermates during a 30 min exposure time period (right). **(B)** Forced swimming test. Schematic of the testing environment (left). Mice are placed in an inescapable cylinder filled with water and the time spent floating (immobile) is measured. Mice were exposed to the forced swim test for 6 min on two consecutive days. HCN3^−/−^ animals tended to spend more time floating on the second testing day (right). All values are Mean ± SEM, ^**^p < 0.01.

We also tested for motor stereotypies, such as repetitive grooming, jumping, and digging, using the marble-burying assay (Figure 6A). For this test, a cage is prepared with a defined layer of wood chip bedding and marbles are placed in a regular pattern on top of the bedding surface. An animal is placed in the cage for 30 min. Thereafter, the mouse is removed, and the number of marbles buried with bedding is counted. Within the 30-min observation period, HCN3^−/−^ mice buried a significantly higher number of marbles than wild type (*t*_18_ = 2.933; *p* < 0.01; unpaired *t*-test; Figure 6A, right). Since we failed to observe changes in anxiety-related behavior on an elevated plus-maze or in the light-dark box (see Figure 5) and general arousal (as assessed by novelty-induced grooming; Figure 5H), it is difficult to interpret this finding.

Depressive-like behavior was tested using the forced swimming test (Figure 6B). This test measures the time spent swimming vs. the time spent floating in a tall cylinder filled with water, from which the mice cannot escape. After some time the animal may stop swimming and begin floating. This incidence of passive stress coping is regarded as a sign of behavioral despair. For the test, active (swimming and attempts for climbing) and passive (immobility) behavior is determined and the fraction of floating behavior characterized by passive immobility in relation to total observation time is calculated. Both, wild type and HCN3^−/−^ mice showed an increase in floating from the first to the second exposure, however, without significant genotype differences [d1–GT: *F*_(1, 38)_ = 0.1583, *p* > 0.05; d2–GT: *F*_(1, 38)_ = 2.927, *p* > 0.05; 2-way ANOVA for repeated measures; Figure 6B].

**Figure 7:**
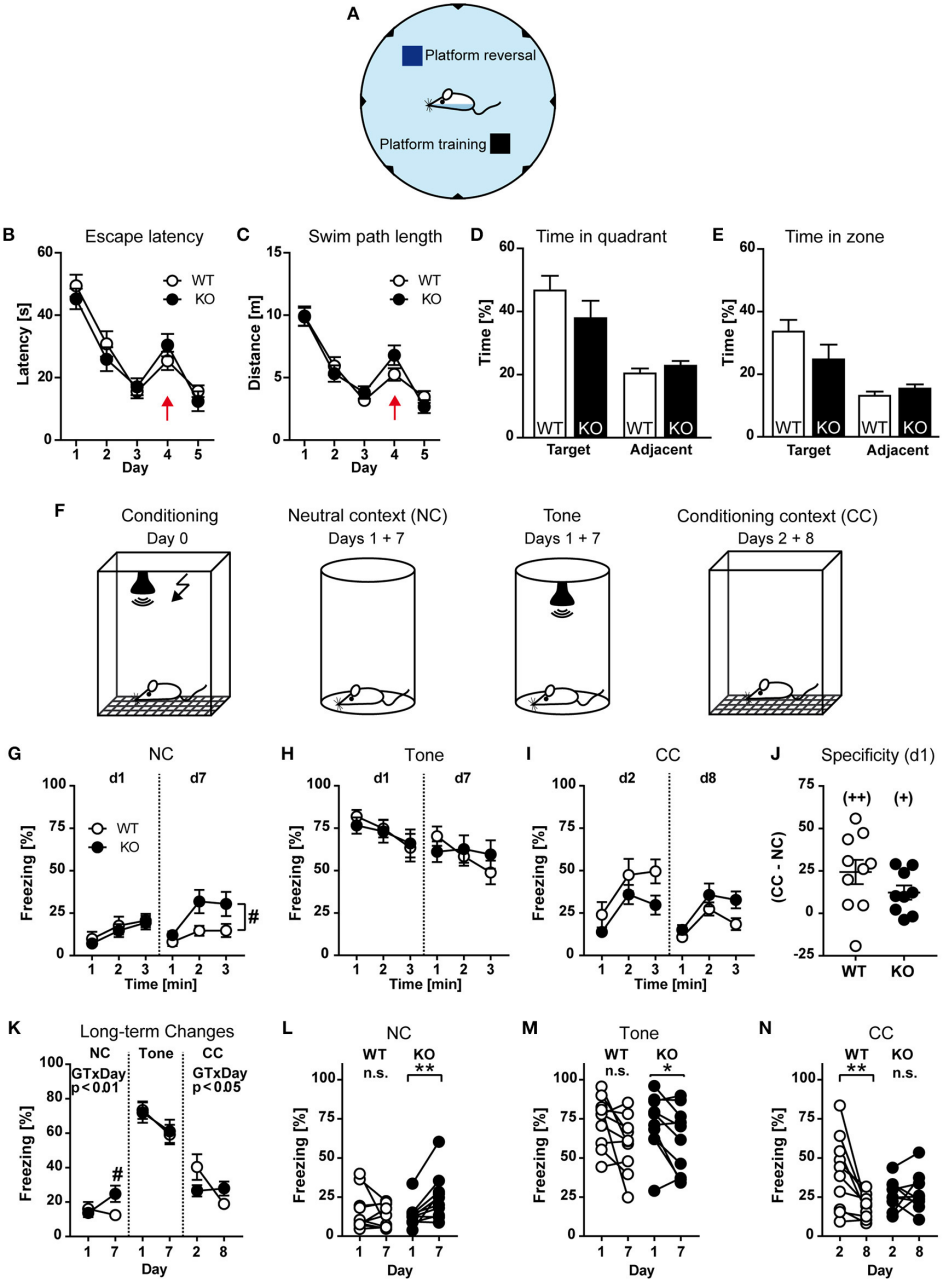
Behavioral characterization of HCN3^−/−^ mice (part 3). **(A–E)** Morris water maze. During training (days 1-3) the platform was hidden in a specific quadrant of the maze and its position was changed on the first trial of day 4 (probe trial) to a different quadrant and remained there until the end of the experiment **(A)**. According to escape latencies **(B)** and swim path length **(C)**, mice showed robust learning (days 1–3), reversal effect (day 4; indicated by red arrow), and re-learning (day 5) with no evidence for a genotype effect. During the probe trial (first trial on day 4) the mice showed a robust preference for the trained quadrant compared to the average of the two adjacent quadrants **(D)** and for a circular zone (12.5% of pool surface) drawn around the former platform location **(E)**. **(F–N)** Fear conditioning. For conditioning, mice were placed into a shock chamber and a tone was presented followed by an electric foot shock (**F**, first panel). On d1 and d7 after conditioning, mice were observed in the neutral context before (NC) and during subsequent exposure of the tone (tone) for 3 min each, and to the shock chamber (CC) on d2 and d8 without applying an electric shock or a tone (3 min each). **(G)** Freezing in the neutral context during the 3 min before tone presentation (expressed in 1-min intervals) at d1 and d7. **(H)** Freezing to the tone at d1 and d7 (1-min intervals). **(I)** Freezing in the conditioning context at d1 and d7 (1-min intervals). **(J)** Specificity of contextual fear expressed as the difference between freezing in the CC and the NC. **(K)** Long-term changes in freezing from d1 to d7. **(L–N)** Individual data plots depicting long-term changes in freezing from d1 to d7 separately per genotype. ^*^p < 0.05, ^**^p < 0.01 (paired *t*-test), ^+^*p* < 0.05, ^++^*p* < 0.01 (one-sample *t*-test against theoretical value 0), ^#^*p* < 0.05 (KO vs. WT; 2-way ANOVA followed by Bonferroni *post-hoc* test, if appropriate). One KO was excluded from further analysis of contextual fear since it was identified as outlier (http://www.graphpad.com/quickcalcs/grubbs1/).

Finally, cognitive functions such as learning and memory were tested using the Morris water maze and fear conditioning. To assess functional implications of HCN3 knock-out on spatial memory, we tested mice of both genotypes in the hidden platform version of the Morris water maze (Figure 7A). Mice were trained to locate to a hidden escape platform in a water basin on three consecutive days (navigation training). On day 4 and 5 the reverse hidden platform learning task was performed by moving the platform to the diagonally opposite quadrant.

During water maze training, the parameters escape latencies and swim path length indicate robust learning, reversal effect, and re-learning in both groups of mice, with no difference between the genotypes (Figures 7B,C). There was also no difference in swim speed between the two groups of mice [Genotype: *F*_(1, 28)_ = 2.189, *p* > 0.05; Supplementary Figure 5], or in any of the other parameters assessed, such as incidence of floating and wall approaches (data not shown). Overall the mice showed little wall hugging, with mutants perhaps tending to spend somewhat more time near the wall [Genotype: *F*_(1, 28)_ = 3.522, *p* > 0.05; Supplementary Figure 5]. During the probe trial on day 4, mice showed strong preference for the trained quadrant [Place: *F*_(1, 28)_ = 21.34, *p* < 0.001; Figure 7D]. This was calculated by comparing the time spent in the trained quadrant vs. the averaged time the animals spent in the two adjacent quadrants. Since, during the probe trial, the hidden platform is relocated to the opposite quadrant, it cannot serve as a neutral control and therefore was not included into the calculation. The preference of HCN3^−/−^ mice for the training quadrant was indistinguishable from that of wild type mice [Place × Genotype: *F*_(1, 28)_ = 0.202, *p* > 0.05; Figure 7D]. In addition, mice showed a clear preference for a circular zone drawn around the former goal location (12.5% of pool surface) during the probe trial [Place: *F*_(1, 28)_ = 16.48, *p* < 0.01; Figure 7E], again with no statistical significant difference between the two genotypes [*F*_(1, 28)_ = 1.993, *p* > 0.05; Figure 7E]. In summary, the water-maze place navigation task revealed robust learning, reversal effect, re-learning and probe trial retention in both groups, with no significant genotype differences, indicating that HCN3 channels are not necessary for these processes.

For fear conditioning (Figure 7F), HCN3^−/−^, and wild type mice were placed into a shock chamber and after 3 min (pre-training) a tone was presented for 30 s. During the last 2 s an electric foot shock was applied simultaneously. On day one after conditioning, HCN3^−/−^ and wild type animals were placed in a novel chamber representing the neutral context (NC) and were observed for 3 min (Figure 7F, second panel). Subsequently a tone, but no shock was applied and the mice were observed for additional 3 min (Figure 7F, third panel). On day two after conditioning, HCN3^−/−^ and wild type mice were exposed to the shock chamber (Conditioning context, CC) without applying an electric shock or a tone (Figure 7F, fourth panel). These exposures to neutral and conditioning context were repeated seven days after the first exposure (i.e., on day 7 and 8 after conditioning). Freezing behavior served as a measure of fear. During the 3 mins of pre-training, before presentation of the tone and foot shock, both genotypes exhibited very low and statistically indistinguishable levels of immobility [GT: *F*_(1, 18)_ = 0.7615, *p* > 0.05; GT × Time: *F*_(2, 36)_ = 1.034, *p* > 0.05; 2-way ANOVA for repeated measures; data not shown]. Furthermore, HCN3^+/+^ and HCN3^−/−^ showed the same low levels of freezing during the 3 min before tone presentation in the novel (neutral) context at d1 [GT: *F*_(1, 18)_ = 0.2399; *p* > 0.05; GT × Time: *F*_(2, 36)_ = 0.0592, *p* > 0.05; Figure 7G]. During re-exposure to the neutral context at d7, however, HCN3^−/−^ showed significantly more basal freezing [GT: *F*_(1, 18)_ = 5.427; *p* < 0.05; Figure 7G]. Presentation of the tone resulted in a strong increase in freezing, both at day 1 and 7, which was indistinguishable between the two genotypes [Figure 7H; Genotype: *F*_(1, 18)_ ≤ 0.047, *p* ≥ 0.830]. There were also no statistically significant differences in contextual fear (conditional context, without tone presentation or shock application) both at d2 [GT: *F*_(1, 17)_ = 2.692; *p* > 0.05] and d8 [GT: *F*_(1, 17)_ = 3.792; *p* > 0.05; Figure 7I]. In addition, both HCN3^+/+^ and HCN3^−/−^ showed the same specificity of contextual fear (expressed as the difference in freezing in the conditioning context and the neutral context; *t*_17_ = 1.415, *p* > 0.05; unpaired *t*-test; Figure 7J).

On closer inspection of short-term and long-term changes in freezing from d1 to d7 and d2 to d8, respectively, we observed a lack of fear adaption over the course of tone presentation in HCN3^−/−^ compared to HCN3^+/+^ at d7 [*F*_(2, 36)_ = 4.051, *p* = 0.025], but not at d1 [*F*_(2, 36)_ = 0.506, *p* = 0.606], as well as striking genotype differences in case of freezing in the neutral context before tone presentation (GT × Day: *F*_(1, 17)_ = 8.856, *p* < 0.01; Figure 7K) and in the conditioning context [GT × Day: *F*_(1, 17)_ = 7.989; *p* < 0.05; Figure 7K], but not freezing to the tone [GT × Day: *F*_(1, 18)_ = 0.1909; *p* > 0.05; Figure 7K]. To emphasize the long-term extinction at the level of individual animals, subsequent analyses separately per genotype confirmed a significant increase in freezing in the neutral context in HCN3^−/−^ (*t*_9_ = 3.564, *p* < 0.01; paired *t*-test; Figure 7L), but not HCN3^+/+^ mice (*t*_9_ = 0.938, *p* > 0.05; Figure 7L), whereas freezing to the tone was decreased in HCN3^−/−^ and tended to be decreased in HCN3^+/+^ animals (Figure 7M). In contrast, HCN3^+/+^ (*t*_9_ = 3.205, *p* = 0.01; Figure 7N), but not HCN3^−/−^ (*t*_8_ = 0.329, *p* > 0.05; Figure 7N), showed a significant decrease in contextual fear from d2 to d8. In summary, HCN3^−/−^ showed deficits in acute fear adaption upon re-exposure to the tone and long-term processing of contextual fear, which can be interpreted as lack of extinction and increase in context generalization.

## Discussion

The functional role of HCN1 and HCN2 channels have been extensively analyzed in the heart and the brain. While for all members of the HCN channel family genetic knockout mouse models are available, the neuronal phenotype of HCN3^−/−^ mice has not been investigated and reported so far. In this study, we have used HCN3^−/−^ mice to investigate the specific physiological role of this channel in the CNS. Here, we specifically focused on the significance of this channel for circadian rhythm and behavior.

Using the HCN3^−/−^ mouse line, we have previously reported the role of HCN3 channels for late repolarization in action potential of ventricular cardiomyocytes (Fenske et al., 2011). A comprehensive characterization of the HCN3^−/−^ mouse revealed that there are no obvious other functional organ specific phenotypes. In particular liver function tests, exocrine, and endocrine pancreatic function, homeostasis of electrolytes and regulation of plasma lipid and lipoprotein levels were normal. Also, body weight was unaffected. However, we found that Ca^2+^ and Urea levels were significantly decreased in HCN3^−/−^ animals. Since HCN3 is expressed in kidneys of several species, the question arises whether these findings could represent a renal phenotype, or might be caused by extra-renal metabolic or humoral changes. This issue needs to be addressed in future studies. Furthermore, we did not find any evidence for a functional role of these channels for the regulation of hypothalamus-dependent release of corticotropin-releasing factor, and corticosterone. This indicates that HCN3 channels do not seem to be functionally relevant for regulation of brain CRF or plasma glucocorticoid levels under baseline conditions. Nevertheless, due to their high expression in the PVN, HCN3 channels might still be implicated in the stress response provoked by acute or chronic stressors, which needs to be verified in future experiments. Furthermore, there was no change in ADH dependent electrolyte or water balance, indicating that HCN3 channels in ADH positive cells in the hypothalamic region are not critical for regulating plasma electrolyte composition under baseline conditions. We then focused in particular on the role of HCN3 in the circadian system and in the control of behavior.

### Circadian system

To analyze the function of HCN3 channels in the major network regulating the circadian rhythm (Figure 1) we studied expression and function of this channel in the SCN, the retina, and in the IGL. Surprisingly there is no clear expression of HCN3 in the SCN. Our own results as well as data from the literature (Notomi and Shigemoto, 2004) suggest that in the SCN, other HCN channels may be present, most likely HCN4. The role of this channel in controlling circadian behavior is not known.

In the retina, HCN3 channel labeling was restricted to the outer and inner plexiform layers of the retina, which harbor the synapses of photoreceptor and bipolar neurons, respectively. This finding is in line with a previous report, which also found HCN3 expression in the outer plexiform layer (Muller et al., 2003). The authors of this study concluded that HCN3 is anatomically restricted to the base of cone pedicle in the outer plexiform layer and suggested that HCN channels might directly modulate synaptic transmission of photoreceptor activity to bipolar cells (Muller et al., 2003), which was indirectly tested in the present study. Surprisingly, our electrophysiological results did not confirm this appealing hypothesis. In fact, retinal function testing in HCN3^−/−^ animals indicated normal cone and rod function under both photopic and scotopic conditions. In particular, the b-wave was normal in HCN3^−/−^ mice, which directly reflects the activity of bipolar cells in the inner retina and indirectly reflects activity of photoreceptors and successful synaptic transmission at the first synapse. A normal, unreduced b-wave therefore indicates regular activity in these synapses and argues against a major role of HCN3 channels for transmission in photoreceptor synapses including those of cone pedicles. Given the relatively large deactivation time constants of HCN3 channels (Fenske et al., 2011) it would be possible that HCN3 channels are involved in slower steps of visual information processing in the retina. One possible scenario would be that HCN3 is involved in long-term adaptation and thus complement the role of HCN1 channels in short-term adaptation of visual function. HCN1 channels have been shown to be predominantly located in the inner segments of rod and cone photoreceptors (Seeliger et al., 2011). These channels prevent saturation of the retinal network following bright light exposure, prevent prolonged “blinding” by light and speed up regain of vision. Beside HCN1 and HCN3 channels, HCN4 channels are present in the retina (Muller et al., 2003). The function of this channel is not known. In conclusion, retinal function is completely normal in HCN3^−/−^ mice. Furthermore, the flow of photic information from the retina to the SCN and IGL via the retinohypothalamic tract, which is critical for resetting of the circadian clock, is also normal, even in the absence of HCN3.

We found high levels of HCN3 protein expression in the IGL. This finding is in line with reports from Ying et al. (2011). In addition, we find evidence for different populations of neurons in the IGL, one population in which I_h_ is completely absent in HCN3^−/−^ neurons, and a second population in which a remaining I_h_ is present. Consistent with our IHC experiments this remaining I_h_ is most likely conferred by HCN2. The IGL has been demonstrated to be an important nucleus upstream of the SCN, which integrates photic and non-photic information (Delogu et al., 2012) and eventually implicates circadian behavior via output firing to the SCN. In contrast to these hypotheses, our results indicate that HCN3 channels do not play a major role in the regulation of circadian locomotor behavior under constant lighting conditions. Furthermore, we could not confirm a major role of these channels for light induced resetting mechanism of the circadian clock. Moreover, HCN3 channels do not play an important role in the speed of adaptation in the behavior of mice to a new LD cycle. However, in the absence of a light cue [constant darkness conditions (DD)] HCN3^−/−^ animals display less activity (Figure 4C). This result suggests that HCN3 is important for reinforcing locomotor activity under DD conditions. Together the circadian experiments indicate that even if the HCN3 component of I_h_ is responsible for rhythmic oscillations formed by spontaneous low threshold spike burst firing of pacemaker neurons in the IGL as suggested by Ying et al., this change is without consequence for IGL dependent modulation of the SCN and SCN dependent circadian behavior in the presence of light cues. This may be different in the absence of light cues as observed under DD conditions. In conclusion, although HCN3 channels are highly expressed in the IGL, their deletion does not significantly alter photic, IGL-specific regulation of circadian function. However, we cannot completely rule out the possibility that in HCN3^−/−^ animals the compensatory upregulation of other HCN channel family members might mask a possible phenotype. Given that in (Fenske et al., 2011) no such upregulation was found in microarray experiments this possibility is rather unlikely.

### Behavior

Given the broad intracerebral expression of the HCN3 channel, behavioral consequences of its functional knock-out were remarkably distinct: Except for a reduction in horizontal locomotion, we failed to observe genotype effects in a variety of tests assessing motor learning and motor coordination (rotarod), exploration-based anxiety-related behavior (elevated plus maze, dark-light transition test), behavioral stress coping (forced swim test), and hippocampus—(Morris water maze, acquisition, and consolidation of contextual fear) and amygdala-dependent learning (auditory-cued fear conditioning). In contrast, HCN3^−/−^ showed enhanced marble burying and impaired processing of contextual fear upon repeated (re)exposure to the conditioning and the neutral test context: specifically, whereas wild type mice showed extinction of contextual fear from d2 to d8, HCN3^−/−^ failed to do so. In contrast, HCN3^−/−^, but not wild type mice, developed generalized fear to the neutral context from d1 to d7. Here, the process of picking up of the animals by the tail itself and placing them into the test environment might be sufficient to trigger “context generalization.” At the same time, we cannot rule out whether the failure to decrease freezing behavior might be ascribed to second-order conditioning. In this type of conditioning, the animals learn to associate the neutral context with the aversive tone during testing at day 1. Alternatively, an unspecific increase in general anxiety could explain the behavioral effects. In contrast, the observed alterations cannot be explained by general changes in locomotor activity or long-term habituation, since both genotypes showed the same decrease in locomotion over two consecutive exposures to an open field. Another factor that might influence the outcome of the fear conditioning paradigm are changes in auditory function. While we did not perform in depth analysis of the auditory function in HCN3^−/−^ mice, we can exclude a major overall auditory phenotype in these mice, because freezing behavior upon presentation of the tone was similar in wild type and HCN3^−/−^ animals. This finding indicates that even though we cannot rule out subtle differences in auditory function between HCN3^−/−^ and WT mice, these differences, if present, do not interfere with the fear conditioning paradigm and our conclusions. Finally, a change in pain perception *per se* could account for changes in fear conditioning. It has been shown that HCN channels are expressed in dorsal root ganglia of mice and rat (Kouranova et al., 2008; Hou et al., 2015; Usoskin et al., 2015). There is also evidence that HCN2 channels might be involved in the regulation of neuropathic and inflammatory pain (Emery et al., 2011). By contrast, there is currently no evidence that HCN channels are involved in acute somatosensory nociceptive pain. In line with this notion, the study by Emery et al. (2011) showed that pain reactions toward acute mechanical and heat stimuli were not affected after application of ZD7288, a non-selective blocker of HCN channels. This result indicates that even though HCN3 channels might be expressed in dorsal root ganglia, these channels are not involved in fundamental regulation of acute somatosensory nociceptive pain, e.g. the subtype of pain relevant in fear conditioning experiments.

Currently we can only speculate about the biological processes underlying the increase in marble burying and fear generalization and the impairment in fear extinction. Given (i) the close correlation between pharmacologically enhanced monoamine levels within the mPFC and alterations in marble burying (increased levels of serotonin correlated positively with inhibition of marble burying, whereas changes in noradrenaline revealed the reverse relationship; (Kobayashi et al., 2008), (ii) the role of the mPFC in fear extinction (for review see Maren et al., 2013), and (iii) the implication of mPFC projections to the thalamus in generalization of contextual fear (Xu and Sudhof, 2013), it is tempting to assume a contribution of corticothalamic projections to the observed phenotype. In line with this hypothesis, HCN channels seem to be present in dendritic spines of mPFC neurons (Wang et al., 2007). Furthermore, HCN3 channels are expressed in the basolateral amygdala and the hippocampus in the rat (Notomi and Shigemoto, 2004) and mouse brains (own observations). These brain regions are implicated in the formation of associative memory, learning and expression of fear. On the cellular level, HCN channels have been associated with a variety of physiological roles in the CNS, such as setting of the resting membrane potential and therefore controlling the excitability of a given neuron, or synaptic integration and plasticity. Hence, deletion of HCN3 might alter the intrinsic activity of neurons in the amygdala complex or hippocampal neurons and disturb the proper information processing of learned fear. Future studies will reveal the details how exactly HCN3 channels in these neurons are involved in regulation of fear generalization.

## Author contributions

MS performed experiments, analyzed data and wrote the manuscript. SF, VH, and EB provided guidance and designed experiments. VS, MS-W, RM, and JD performed experiments and analyzed data. JED and UA planned and performed experiments, analyzed data, and helped to write the manuscript. DW planned and performed experiments and analyzed data, MWS planned experiments, analyzed data and assisted in writing the manuscript. CW and SM provided guidance, analyzed data and assisted to write the manuscript. MB carried out the study design, provided guidance and assisted to write the manuscript. CW-S carried out the study design and wrote the manuscript. All authors have read and approved the final version of the manuscript.

### Conflict of interest statement

The authors declare that the research was conducted in the absence of any commercial or financial relationships that could be construed as a potential conflict of interest.
