# Supplementary material for: Disturbed Processing of Contextual Information in HCN3 Channel Deficient Mice
Source: Front Mol Neurosci. 2018 Jan 9;10:436. doi: 10.3389/fnmol.2017.00436 (PMC5767300; doi:10.3389/fnmol.2017.00436)
Supplement: Supplementary file 1 [file DataSheet1.DOCX]

Supplementary Material

Disturbed processing of contextual information in HCN3 channel deficient mice

**Marc S. Stieglitz, Stefanie Fenske, Verena Hammelmann, Elvir Becirovic, Verena Schöttle, James E. Delorme, Martha Schöll-Weidinger, Robert Mader, Jan Deussing, David P. Wolfer, Mathias W. Seeliger, Urs Albrecht, Carsten T. Wotjak, Martin Biel, Stylianos Michalakis, and Christian Wahl-Schott**^*^.

*** Correspondence:** Corresponding Author: Christian.Wahl@cup.uni-muenchen.de


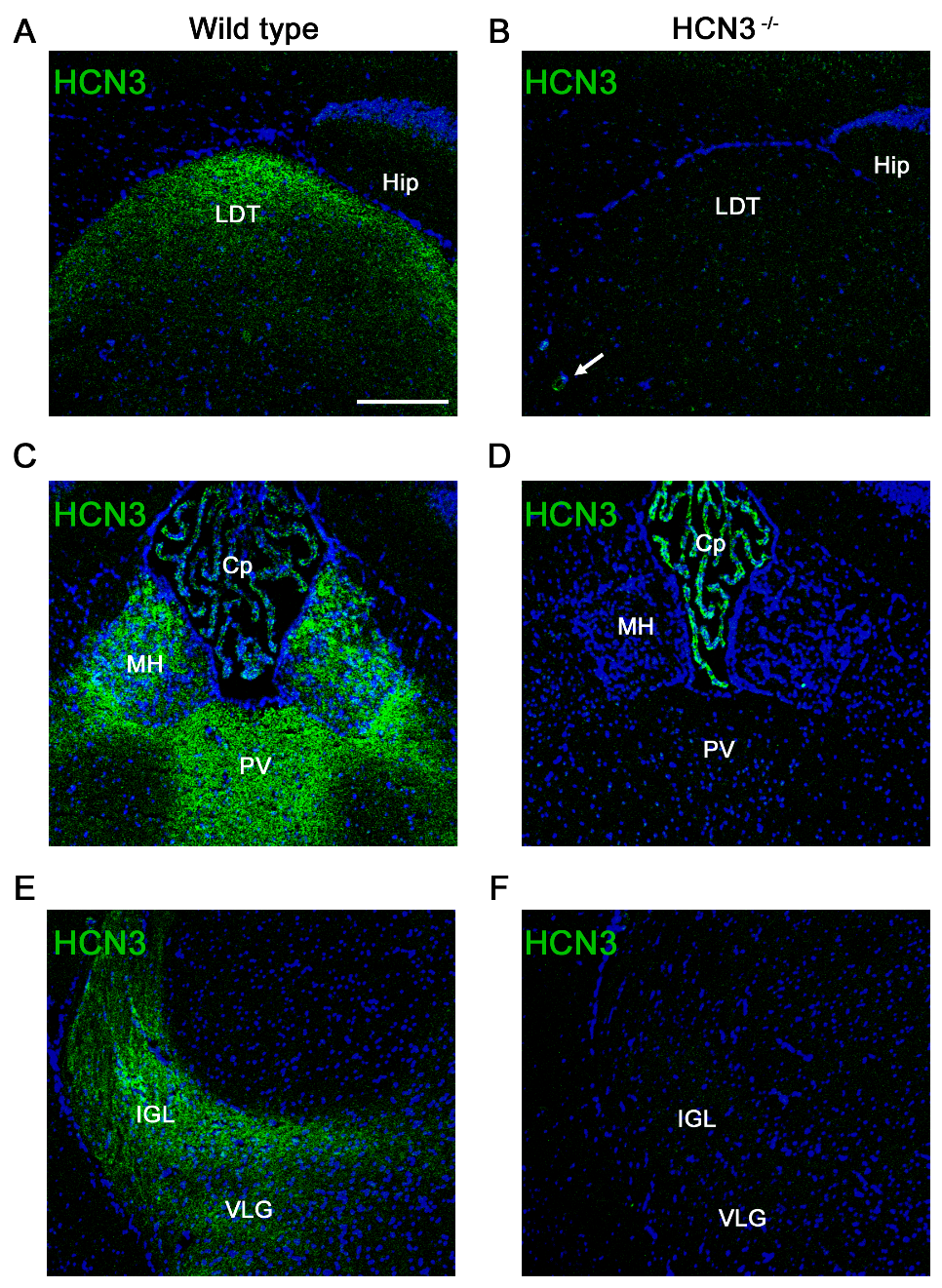


**Supplementary Figure 1. HCN3 expression in thalamic regions and specificity of the antibody. (A)** HCN3 channel proteins (green) in the thalamic nucleus laterodorsalis (LDT) of wild type animals. **(B)** The specific HCN3 staining is absent in HCN3^-/-^ animals. The arrow indicates the unspecific staining of a blood vessel. **(C)** HCN3 channel protein is found in the medial habenula (MH) and the paraventricular nucleus of the thalamus (PV). **(D)** The specific HCN3 staining is absent in HCN3^-/-^ animals, however, in the choroid plexus (Cp) the antibody shows unspecific binding. **(E)** HCN3 protein is found in the intergeniculate leaflet (IGL) and ventral geniculate nucleus (VLG). **(F)** The HCN3 signal is absent in HCN3^-/-^ animals; HCN3: green; Hoechst (nuclei): blue; Scale bar = 200 µm; Hip: hippocampus


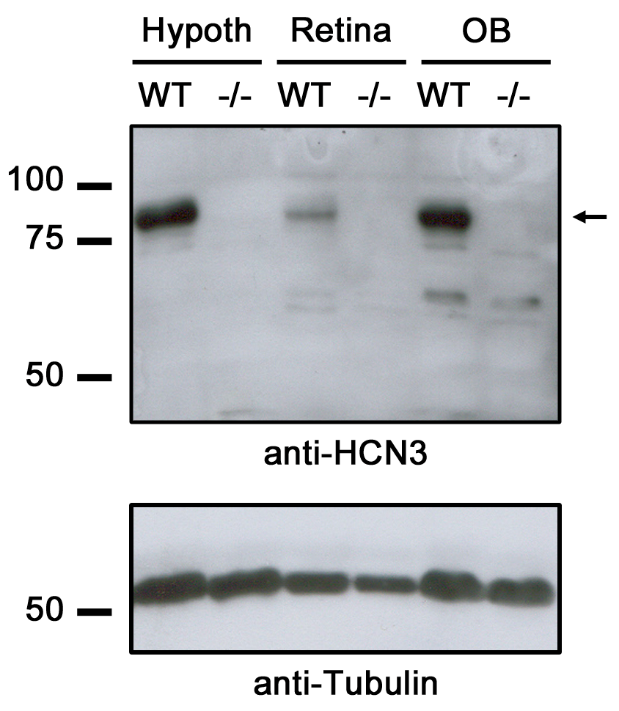


**Supplementary Figure 2. Western blot of HCN3 protein.** Upper panel: Western blot against HCN3 (rabbit anti-HCN3, 1:2000) of homogenates from mouse hypothalamus (hypoth), retina and olfactory bulb (OB). Lower panel: the same blot probed with anti-tubulin antibody (mouse anti-tubulin, 1:400). The arrow indicates the height of the expected HCN3 band.


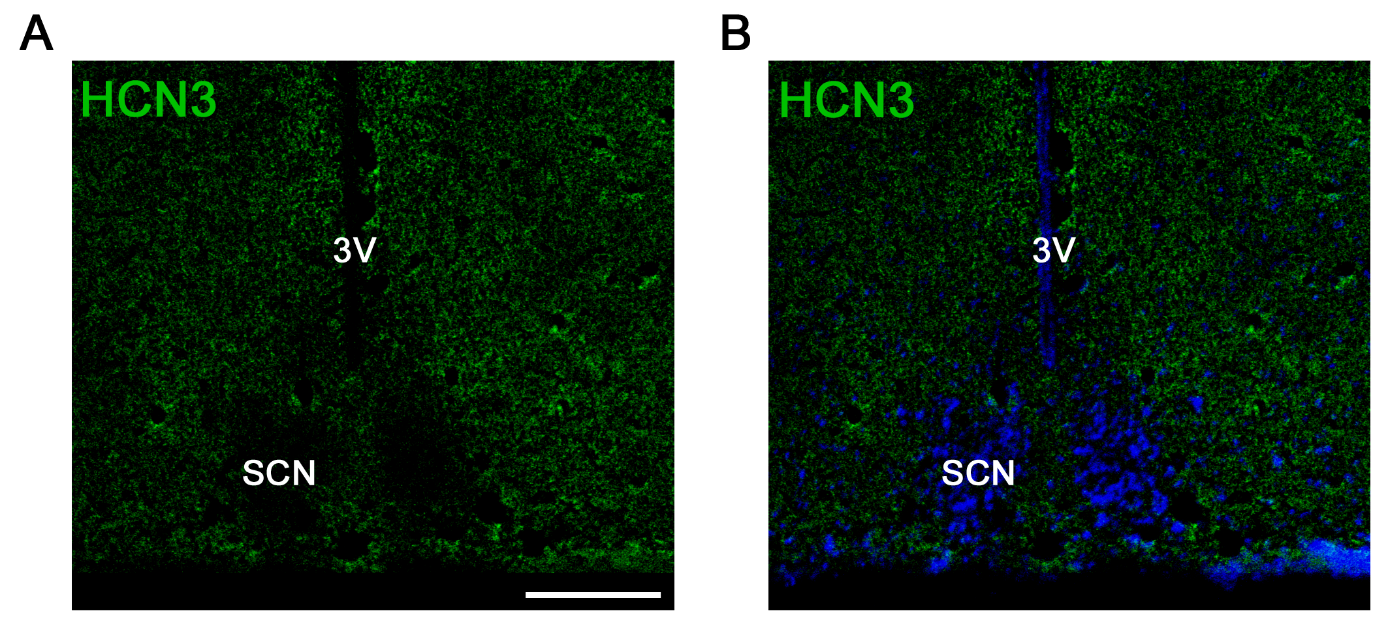


**Supplementary Figure 3. HCN3 cannot be found in the suprachiasmatic nucleus in immunohistochemical experiments. (A)** HCN3 staining (green) is found in the hypothalamus, however, it spares the suprachiasmatic nucleus (SCN). **(B)** Overlay of HCN3 (green) and nucleic staining with Hoechst (blue); scale bar = 200 µm; 3V: third ventricle

**Role of HCN3 channels in the hypothalamic control of corticosterone, fluid and electrolyte homeostasis.**

HCN3 channels are expressed in specific nuclei of the hypothalamus (Nucl. paraventricularis, Nucl. suprachiasmaticus, Nucl. dorsomedialis, Nucl. lateralis, Nucl. hypothalamicus anterior and Nucl. preopticus medialis; Table 2). Given that the hypothalamus is the coordinating center of homeostatic and metabolic functions including endocrine regulation, we were wondering whether HCN3 is functionally relevant for these functions. We found that HCN3 channels are present in the hypothalamic-pituitary-adrenal axis of the endocrine system. In IHC experiments specific immunelabelling of HCN3 was detected in the paraventricular nucleus (PVN) of the hypothalamus, while staining of other HCN channel subtypes were only present at very low levels (Supplementary fig. 4A, B). Furthermore, HCN3 transcripts are present in the pituitary and adrenal glands (Supplementary fig. 4C). While this specific expression pattern suggests a potential function in the hypothalamic-pituitary-adrenal axis, brain CRF and plasma corticosterone levels were normal at all time points assessed (Supplementary fig. 4D, E).

**
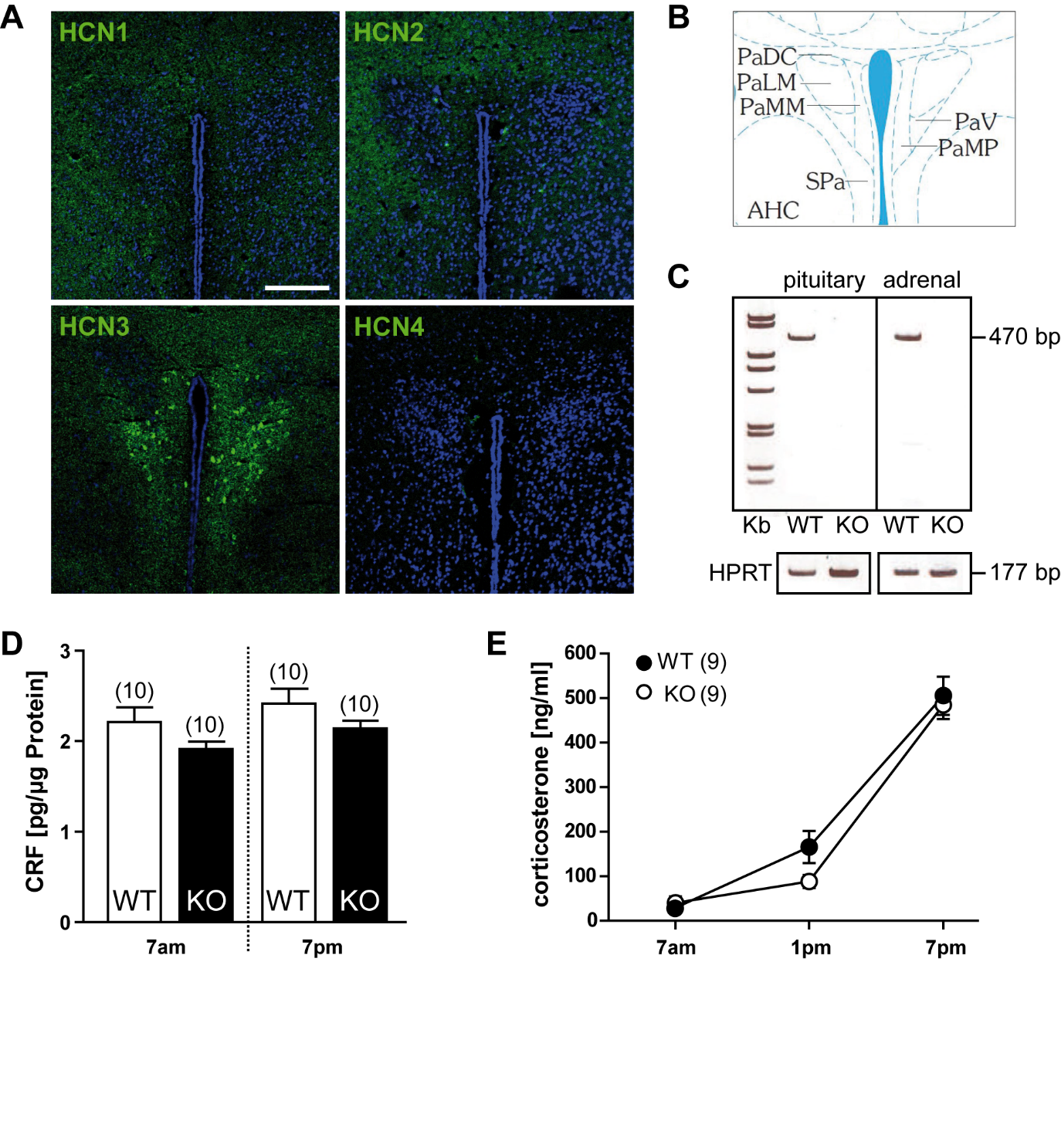
**

**Supplementary Figure 4. HCN3 channels are present in the hypothalamic-pituitary-adrenal axis. (A)** Expression of HCN1-4 channel proteins in the paraventricular nucleus (PVN): HCN3 is the most prominently expressed HCN channel isoform in the PVN (green: HCN1-4 respectively, blue: Hoechst). **(B)** Schematic of the PVN anatomy; Adapted from: Franklin&Paxinos, The mouse brain in stereotaxic coordinates, Elsevier, 2007. PaDC: paraventricular nucleus dorsal cap; PaLM: paraventricular nucleus lateral magnocellular part; PaMM: paraventricular nucleus medial magnocellular part; PaV: paraventricular nucleus ventral part; PaMP: paraventricular nucleus medial parvicellular part; SPa: subparaventricular zone of the hypothalamus; AHC: anterior hypothalamic area central part. **(C)** Reverse-transcription polymerase chain reaction analysis of HCN3 expression in the pituitary (left) and adrenal glands (right): HCN3 (470 bp) is expressed in WT but not HCN3^-/-^ glands; HPRT (Hypoxanthine-guanine phosphoribosyltransferase; loading control) **(D)** Plasma CRF levels detected by ELISA testing. CRF levels were similar in WT and HCN3^-/-^ animals. **(E)** Corticosterone plasma levels detected by radio-immune-assay (RIA). Corticosterone levels determined at three different time points were similar in WT and HCN3^-/-^ animals.


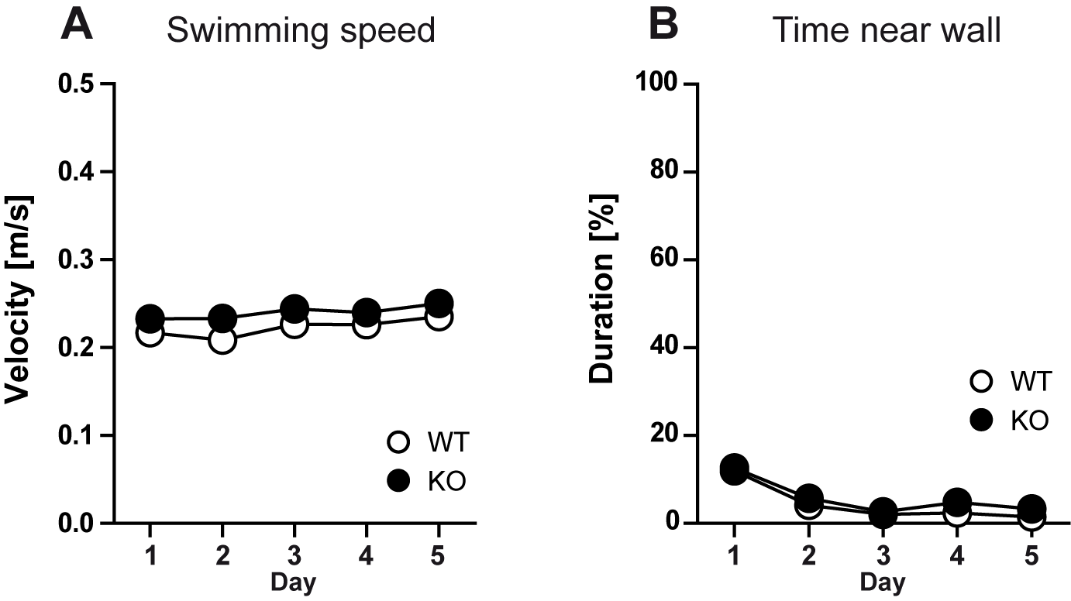


**Supplementary Figure 5. Morris water maze. (A)** Swimming speed in the morris water maze was unaffected by the genotype and similar on all testing days. **(B)** The relative time the animals spent near the wall was similar in wild type (WT) and knockout (KO) animals.
